# Modeling the spread of polio in an IPV-vaccinated population: lessons learned from the 2013 silent outbreak in southern Israel

**DOI:** 10.1186/s12916-016-0637-z

**Published:** 2016-06-23

**Authors:** Rami Yaari, Ehud Kaliner, Itamar Grotto, Guy Katriel, Jacob Moran-Gilad, Danit Sofer, Ella Mendelson, Elizabeth Miller, Amit Huppert, for the POG group, E. Anis, E. Kopel, Y. Manor, O. Mor, L. Shulman, R. Singer, M. Weil

**Affiliations:** Bio-statistical Unit, The Gertner Institute for Epidemiology and Health Policy Research, Chaim Sheba Medical Center, Tel Hashomer, 52621 Israel; Biomathematics Unit, Department of Zoology, Faculty of Life Sciences, Tel Aviv University, 69978 Tel Aviv, Israel; Public Health Services, Ministry of Health, Jerusalem, Israel; Faculty for Health Sciences, Ben-Gurion University of the Negev, Beer-Sheva, Israel; Department of Mathematics, ORT Braude College, Karmiel, Israel; Central Virology Laboratory, Ministry of Health, Chaim Sheba Medical Center, Tel Hashomer, Israel; School of Public Health, the Sackler Faculty of Medicine, Tel Aviv University, Tel Aviv, Israel; Public Health England Immunisation, Hepatitis and Blood Safety Department, 61, Colindale Avenue, London, UK

**Keywords:** Polio, Inactivated polio vaccine, Oral polio vaccine, Transmission model, Model fitting, Vaccination strategies

## Abstract

**Background:**

Polio eradication is an extraordinary globally coordinated health program in terms of its magnitude and reach, leading to the elimination of wild poliovirus (WPV) in most parts of the world. In 2013, a silent outbreak of WPV was detected in Israel, a country using an inactivated polio vaccine (IPV) exclusively since 2005. The outbreak was detected using environmental surveillance (ES) of sewage reservoirs. Stool surveys indicated the outbreak to be restricted mainly to children under the age of 10 in the Bedouin population of southern Israel. In order to curtail the outbreak, a nationwide vaccination campaign using oral polio vaccine (OPV) was conducted, targeting all children under 10.

**Methods:**

A transmission model, fitted to the results of the stool surveys, with additional conditions set by the ES measurements, was used to evaluate the prevalence of WPV in Bedouin children and the effectiveness of the vaccination campaign. Employing the parameter estimates of the model fitting, the model was used to investigate the effect of alternative timings, coverages and dosages of the OPV campaign on the outcome of the outbreak.

**Results:**

The mean estimate for the mean reproductive number was 1.77 (95 % credible interval, 1.46–2.30). With seasonal variation, the reproductive number maximum range was between zero and six. The mean estimate for the mean infectious periods was 16.8 (8.6–24.9) days. The modeling indicates the OPV campaign was effective in curtailing the outbreak. The mean estimate for the attack rate in Bedouin children under 10 at the end of 2014 was 42 % (22–65 %), whereas without the campaign the mean projected attack rate was 57 % (35–74 %). The campaign also likely shortened the duration of the outbreak by a mean estimate of 309 (2–846) days. A faster initiation of the OPV campaign could have reduced the incidence of WPV even if a lower coverage was reached, at the risk of prolonging the outbreak.

**Conclusions:**

OPV campaigns are essential for interrupting WPV transmission, even in a developed country setting with a high coverage of IPV. In this setting, establishing ES of WPV circulation is particularly crucial for early detection and containment of an outbreak.

**Electronic supplementary material:**

The online version of this article (doi:10.1186/s12916-016-0637-z) contains supplementary material, which is available to authorized users.

## Background

The Global Polio Eradication Initiative represents a major public health challenge. Since 1988, the number of polio endemic countries had fallen from 125 to just two (Pakistan and Afghanistan) by September 2015 and the annual number of wild poliomyelitis cases dropped to an all-time minimum in 2015 [1]. However, eradication efforts have been endangered by the reintroduction of the virus to a number of formerly polio-free countries. An especially alarming case of reintroduction was the ‘silent outbreak’ in Israel, an inactivated polio vaccine (IPV)-using country that had been free of wild poliovirus (WPV) transmission since 1988, and in which transmission was detected by environmental surveillance (ES) in the absence of paralytic cases [2, 3]. The first indication of this outbreak was given on May 28, 2013, by detection of WPV in a sewage sample taken on April 9, 2013, in Rahat, a Bedouin town in the southern part of Israel. Retrospective analysis of sewage samples indicated the existence of WPV in Israel as early as February 2013 [2, 4]. Sequencing of the virus revealed it to be a wild poliovirus type 1 (WPV1) related to strains found in Egypt at the end of 2012 [5]. Following the detection of WPV1, ES was intensified and stool surveys were conducted in towns where WPV1 was found in the sewage and other towns in Israel [4]. This identified additional positive sewage samples, mainly in the south of Israel, but also in other locations in the center and the north of Israel [4]. The stool surveys revealed transmission to be largely restricted to Bedouin children under the age of 10 in the affected towns [6].

The focus of transmission among Bedouin towns in southern Israel may reflect the earlier introduction of WPV1 into this community, and the facilitation of transmission by their larger household size and greater proportion of children in the population (in 2010, 50 % of the Bedouin population were children under 15, compared to 28 % in Israel as a whole [7]). Life style differences may also be contributory factors. Concentration of transmission in children under the age of 10 reflects their unique vaccination status. In 2005, Israel changed from a sequential schedule of IPV followed by oral polio vaccine (OPV) to exclusive use of IPV. OPV induces a strong gut immunity that limits transmission of the virus, whereas IPV induces humoral immunity that protects an individual from the risk of paralysis but has less impact on viral shedding and hence transmission [8]. However, use of OPV entails a small risk of vaccine-associated paralytic polio for non-immune recipients or their contacts [9]. While the initial IPV doses in a sequential schedule protect recipients from vaccine-associated paralytic polio, spread of OPV to unvaccinated contacts still occurs. For this reason, Israel, as well as other countries that had interrupted WPV transmission, decided to discontinue use of OPV in their vaccination programs.

Immediately following the detection of WPV transmission, the Israeli Ministry of Health launched a nationwide IPV catch-up campaign. Two months later, a national OPV vaccination campaign was launched using bivalent oral polio vaccine (bOPV) containing types 1 and 3. The campaign began in the south of Israel on August 5, 2013, targeting all children under 10. Two weeks later, the campaign was extended to the rest of Israel. In towns with persistent findings of WPV1 in sewage, a second round of bOPV vaccination was initiated 2 months later on October 7. The last stool sample positive for WPV1 was taken on February 19, 2014, and the last positive indication of WPV1 in sewage was on April 3, 2014 [10]. By the end of April 2015, the World Health Organization (WHO) officially declared Israel a polio-free country [10], following a 12 month period without any evidence of polio transmission. Throughout the outbreak, no case of poliomyelitis was reported.

Here we model the transmission of WPV1 in children under the age of ten in the Bedouin population of southern Israel, the epicenter of the epidemic according to ES. By fitting a mathematical model to the data collected in the stool surveys, with conditions set using the results of ES, we obtain posterior distributions for the model parameters. Using the model with the estimated parameter values we reproduce the epidemic dynamics, and evaluate the effectiveness of the OPV campaign. We also investigate alternative vaccination scenarios with a view to informing optimal outbreak control measures in other IPV-using countries that may experience WPV transmission after an importation.

## Methods

### Data

#### Demographic data

The modeled population includes children under 10 in the Bedouin population of southern Israel that had not received OPV as part of their routine vaccination schedule prior to the outbreak, together with the newborns added during the outbreak. According to the Israeli Ministry of Health records, the number of Bedouin children under 10 that were targeted to receive OPV on October 2013 was 57,882. Using a yearly birth rate of 3.5 % [11, 12] in the total population of approximately 220,000 Bedouins living in the south of Israel in 2013, we calculated the estimated size of this population throughout the modeled period (Additional file 1).

#### Stool survey data

There were two stool surveys. The first survey was conducted during July 2013, prior to the beginning of the OPV vaccination campaign. The second survey began on September 2013, after most of the first doses of OPV were already given, and continued at a slow rate, up until June 2014; the sampling procedure has been described previously [13]. Briefly, stool samples were obtained from healthy children and infants attending health care clinics for routine immunizations or health checks, or attending day care centers. Some samples were also obtained from stool specimens submitted to diagnostic laboratories for conditions unrelated to polio, most of which were from adults. Approximately 17 % of the stool samples were repeat samples from individuals who were sampled more than once. We removed all remaining samples from an individual taken after a first positive sample and all negative samples from the same individual taken within 1 month of each other. Testing other options for treating repeat samples (e.g., leaving all repeat samples or keeping just the first negative sample from each individual) demonstrated that this choice had no material effect on the model output. Altogether, after removing repeat samples and samples from individuals above 10, there were 895 samples in the first survey in the Bedouin population, of which 51 were positive for WPV1, and 2609 samples in the second survey, of which 23 were positive. Daily results of the stool sampling were used in the fitting procedure, as described below. These data can be found in their entirety in the supplementary data file (Additional file 2).

The results of the stool surveys within the Bedouin population in southern Israel revealed no positive samples (out of a total of 203 samples) in individuals above the age of 10 (Additional file 1: Figure S1). This result reaffirmed our assessment that these individuals, who were vaccinated with OPV in the past, were likely to have only a minor role in the transmission of WPV1 within the Bedouin community. We therefore focused on modeling the transmission of WPV1 in children under the age of 10 that were not vaccinated with OPV in the past. Within the 0–10 age group, the results of the stool surveys suggest potentially higher prevalence rates in the youngest children (Additional file 1: Figure S1). However, there were no significant differences between yearly age groups to warrant the partitioning of the modeled population into several age groups.

#### Vaccination data

The IPV catch-up campaign increased the coverage of individuals vaccinated with at least two doses of IPV in the Bedouin population from 93 % by the beginning of June 2013 to 97 % by the end of the campaign (unpublished data from the Israeli Ministry of Health), and targeted individuals of all ages, including adults. In order to simplify our model, we disregarded the negligible portion of individuals not vaccinated with IPV and assumed the whole population up to age 10 were in the same vaccination status at the beginning of the epidemic (i.e., vaccinated with IPV). The first dose of OPV campaign began on August 5 and reached a maximum coverage of 90 %. A campaign of a second dose of OPV began on October 7 and reached coverage of 53 %. Starting from January 2014, OPV was reintroduced into the routine vaccination schedule program in Israel, with a first dose of OPV given at the age of six months and a second dose a year later (in addition to the IPV vaccinations, which are given at the age of 2, 4, 6 and 12 months, and another booster at age seven). Coverage of first and second dose of OPV in the routine vaccination schedule since its reintroduction is around 90 %. The model incorporates the number of individuals vaccinated each day with a first and second dose of OPV in the campaigns or as part of the routine schedule (see below).

#### Sewage surveillance data

The first indication of WPV1 transmission was obtained through ES of sewage in Rahat on the May 28, 2013, from a sample taken on April 9, 2013. A retrospective analysis revealed that WPV1 first appeared in the Rahat sewage in a sample taken on February 6, 2013 [4]. Following the detection of WPV1, ES was intensified to other locations in Israel, including three more Bedouin towns in the south of Israel in which continuous ES was conducted (Ksaifa, Arara and Tel Sheva). The last positive indication of WPV1 in the sewage of any of these towns was on April 3, 2014 [10]. While there is a quantitative aspect to the sewage sampling results (in terms of the number of viral plaque-forming units or WPV1 qRT-PCR cycle threshold values [4]), there is currently no known method to relate these quantities to the prevalence of WPV1 in the sampled community. However, the sewage sampling data was employed in two ways in the model fitting procedure: (1) by helping to set an upper limit for the initiation time of the epidemic (Additional file 1) and (2) by filtering out unrealistic model simulations which qualitatively contradict the results of the sewage sampling (see ‘Inference’ section).

### Transmission model

**Fig. 1:**
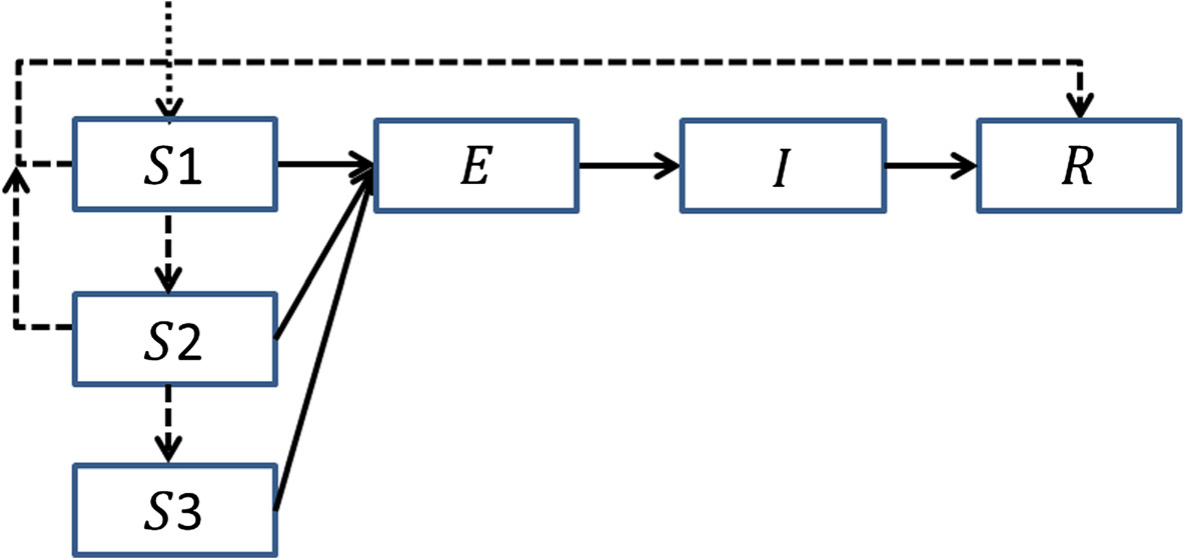
Diagram describing the transmission model compartments. The solid arrows denote the transitions related to infection with wild polio virus. The dashed arrows denote transitions related to vaccination with oral polio vaccine (OPV). The dotted arrow entering the group S1 denotes births. Individuals in the population can be in one of four general states: susceptible (S), exposed (E), infectious (I) or recovered (R). In addition, susceptible individuals are divided into three groups: S1 includes individuals that did not yet receive OPV, S2 includes individuals that received one dose of OPV but remained susceptible, and S3 includes individuals that received two doses of OPV but remained susceptible

A deterministic, discrete-time, susceptible (S) → exposed (E) → infectious (I) → recovered (R) transmission model was used to model the transmission of WPV1 in the population (Fig. 1). In this model, individuals who have been vaccinated with IPV but not OPV lack intestinal immunity and are susceptible to WPV1 infection and, on exposure, can enter the latent period before becoming infectious and finally recovering. Since the transmission of poliovirus is known to be seasonal [14], the reproductive number (a key epidemiological parameter that summarizes the epidemic potential of an infectious disease in a given population [15]) is modeled using a periodic function with three parameters: $\overline{R}$ – the mean reproductive number, *δ* – the amplitude of the seasonal variation in transmission and *ϕ* – the peak time of transmission. The shape of the periodic function was obtained using the results of a study modeling the transmission of WPV in the US during the pre-vaccination era [16], by taking the mean of the estimated seasonal variation in the 10 southern US states that share the same latitudes with Israel (30–33 N) (see Additional file 1 for a full description of the transmission model).

### Vaccination

The model incorporates the number of individuals vaccinated with a first and second dose of OPV on day t, during the two vaccination campaigns and the new routine vaccination program. The vaccinations in the model are protective after a week, as this is the time it takes for the vaccine to build up protective intestinal immunity [17]. The model assumes a per-dose efficacy *ρ*, which implies that an IPV vaccinated individual who is susceptible to infection either becomes completely protected from infection with WPV1 on receipt of a dose of OPV or that the vaccine fails to build protective intestinal immunity after that dose (as in [18–20], for example). For a realistic representation of the effect of OPV on protection against infection the model distinguishes between three susceptible compartments (Fig. 1). The S1 compartment includes individuals who were not vaccinated with OPV, while S2 and S3 include individuals vaccinated with one and two doses of OPV, respectively, for which the vaccine has failed to produce effective immunity. Since the vaccines were given to individuals without knowledge of their actual immune state, some vaccines were given to individuals who were already protected (through infection with WPV1). The model accounts for the probability that a first (second) dose of OPV given on day t is given to a susceptible individual from the S1 (S2) compartment. These probabilities were calculated according to the fraction of the susceptible individuals within the target population for each dose of OPV on day t (see Additional file 1 for more details). Secondary infections with OPV were not included in the model as, with the high vaccine coverage and the restricted transmission capacity of vaccine virus compared to wild virus [21], their effect would be negligible.

### Inference

The results of the stool sampling provide estimates for the prevalence of infection of WPV1 in the population. A stochastic observation model was used to link the prevalence given by the transmission model to the stool survey data, from which a likelihood function was derived, describing the probability of observing the stool survey data given a set of values for the model parameters (see Additional file 1 for details). In addition, the information available from ES was employed in the likelihood function by setting to zero the likelihood of parameter values leading to simulations in which the estimated prevalence after June 2014 (2 months after the last positive findings of WPV in the sewage) was higher than the estimated prevalence during February 6, 2013 (the initial detection time of WPV in the sewage), since higher prevalence after June 2014 would have led to positive findings in the sewage (see Additional file 1 for details).

Altogether, the transmission model employs five parameters with unknown values (Table 1): the mean duration of infectiousness (*d*_*I*_), the mean reproductive number ($\overline{R}$), the amplitude (*δ*) and the peak time (*ϕ*) of the seasonal variation in the transmission and the efficacy of the OPV vaccine (*ρ*). In addition, there is one unknown initial condition which is the initiation time of the epidemic (*t*_0_) (i.e., the introduction time of the first infected case into the population). Markov chain Monte Carlo (MCMC) was used to sample the posterior distribution for the model parameters, defined by the likelihood function and priors, according to the Bayesian methodology [22]. The prior distributions used for the parameters are given in Table 1. The priors for the two seasonality parameters (*δ* and *ϕ*), were set using a normal distribution with the mean and standard deviation of the amplitudes and peak times of the seasonal variation in the 10 southern US states from which the shape of the seasonal function was obtained (Table 1 and Additional file 1). The prior for the per-dose efficacy of OPV (*ρ*) was set using a normal distribution with the estimated mean and standard deviation given in [23]. Parameter values sampled using MCMC were employed with the transmission model in order to obtain 95 % credible intervals (CI) for WPV1 prevalence during the outbreak, as well as the posterior distribution for the overall attack rate and end time of the outbreak.

**Table 1:** List of the transmission model parameters

Parameter	Meaning	Value/prior distribution	Source/ref
*N*	Modeled population size^a^	From 49,692 on September 15, 2012, to 67,248 on December 31, 2014^b^ (Additional file 1: Figure S2)	Israeli Ministry of Health [11, 12]
*d*_*L*_	Mean duration of latent period	4 days	[35, 36]
*d*_*I*_	Mean duration of infectious period	*U* (7,49) days	[8, 25–30]
$\overline{R}$	Mean reproductive number	*U* (1,10)	[37]
*δ*	Amplitude of seasonal variation in transmission^c^	*N* (1,0.414)^d^	[16]
*ϕ*	Peak day of seasonal transmission	*N* (156,17.55)^d^	[16]
*ρ*	Per-dose efficacy of OPV	*N* (0.56,0.23)	[23]
*t*_*0*_	Initiation time of the outbreak	*U* (Sep 15, 2012 to Feb 6, 2013)^e^	[4, 5]

^a^The number of children under 10 in the Bedouin population of southern Israel that were not vaccinated with OPV as part of their routine vaccination schedule prior to the outbreak (including children born after the initiation of the outbreak)

^b^Based on available data from the Israeli Ministry of Health for October 2013 and extended for the whole time period using a birth rate of 3.5 % and a population size of 220,000 for the whole Bedouin population in southern Israel on 2013 (see Additional file 1 for details)

^c^Defined using the normalized mean seasonal variation estimated across 10 southern US states during the pre-vaccine era, so that *δ* = 0 means no seasonal variation while *δ* = 1 means seasonal variation equal to the normalized mean seasonal variation of the southern US states (see Additional file 1 for details)

^d^Based on the variance in the estimates of the seasonal variation in 10 southern US states during the pre-vaccine era (see Additional file 1 for details)

^e^Based on the results of a phylogenetic analysis and the initial finding of WPV1 using ES (see Additional file 1 for details)

## Results

**Fig. 2:**
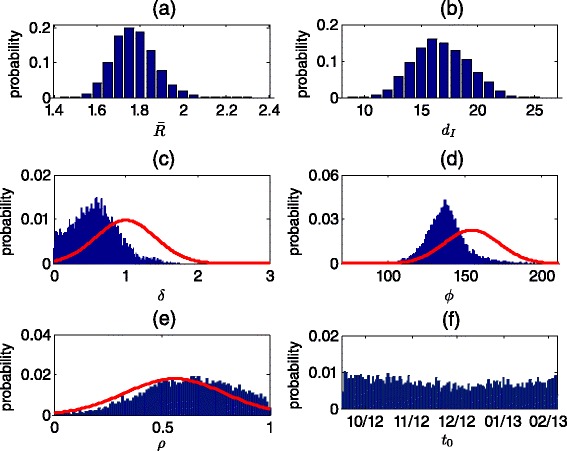
Posterior distributions obtained for the model parameters using MCMC: **a** Posterior distribution for the mean reproductive number. **b** Posterior distribution for the mean infectious period. **c** Posterior distribution for the amplitude of seasonal variation in transmission. The red curve shows the prior distribution based on the variation in the amplitude in 10 southern US states (Table 1 and Additional file 1). The posterior distribution is shifted left from the prior distribution with a mean of 0.57 compared to a mean of 1 in the prior distribution. **d** Posterior distribution for the peak time of seasonal variation in transmission. The red curve shows the prior distribution based on the variation in the peak time in 10 southern US states (Table 1 and Additional file 1). The posterior distribution is shifted left from the prior distribution, with a mean peak day of 138 (May 18) compared to a mean of 156 (June 5) in the prior distribution. **e** Posterior distribution for the per-dose efficacy of OPV. The red curve shows the prior distribution based on [23] (Table 1). The posterior distribution is shifted right from the prior distribution, with a mean efficacy of 0.63 compared to a mean of 0.56 in the prior distribution. **f** Posterior distribution for the start time of the outbreak

Figure 2 shows the posterior distributions obtained for the model parameters and Table 2 gives the summary statistics for these distributions. The posterior distribution of the mean duration of infection (*d*_*I*_) was calculated to have a mean of 16.8 days (95 % CI, 8.6–24.9). The posterior distribution of the mean reproductive number ($\overline{R}$) has a mean of 1.77 (1.46–2.30). The mean peak time of the seasonal variation in transmission (*ϕ*) was found to be May 18 (April 1–August 3), earlier than the estimated mean peak time of June 5 in the 10 southern US states that were used to construct the prior for *ϕ*. The posterior distribution of the amplitude of the seasonal variation (*δ*) was also found to shift left relative to the prior distribution, indicating higher probabilities for a smaller amplitude than the estimated mean in the 10 southern US states. The posterior distribution of the per-dose vaccine efficacy of OPV (*ρ*) was found to shift right relative to the prior distribution with a mean of 0.63 compared to 0.56 in the prior. Regarding the initiation time of the outbreak (*t*_0_), no real information could be inferred from the data, as the whole considered range of values was found to be almost evenly likely.

**Table 2:** Selected results of the model fitting

Output	Mean (95 % CI)
Mean reproductive number	1.77 (1.46–2.30)
Mean duration of the infectious period	16.8 days (8.6–24.9)
Amplitude of seasonal variation in transmission	0.57 (0–1.74)
Peak day of seasonal variation in transmission	138 (91–215) May 18 (Apr 1 to Aug 3)
Per-dose efficacy of oral polio vaccine (OPV)	0.63 (0–1)
Attack rate at the end of 2014 with the OPV campaign	0.42 (0.22–0.65)
Attack rate at the end of 2014 without the OPV campaign	0.57 (0.35–0.74)
Reduction in attack rate due to OPV campaign	0.15 (0–0.40)
End time of the outbreak with the OPV campaign	April 12, 2014 (Jan 19, 2014, to Oct 8, 2014)
End time of the outbreak without the OPV campaign	February 15, 2015 (Feb 18, 2014, to Nov 12, 2016)
Reduction in outbreak duration due to OPV campaign	309 days (2–846)

**Fig. 3:**
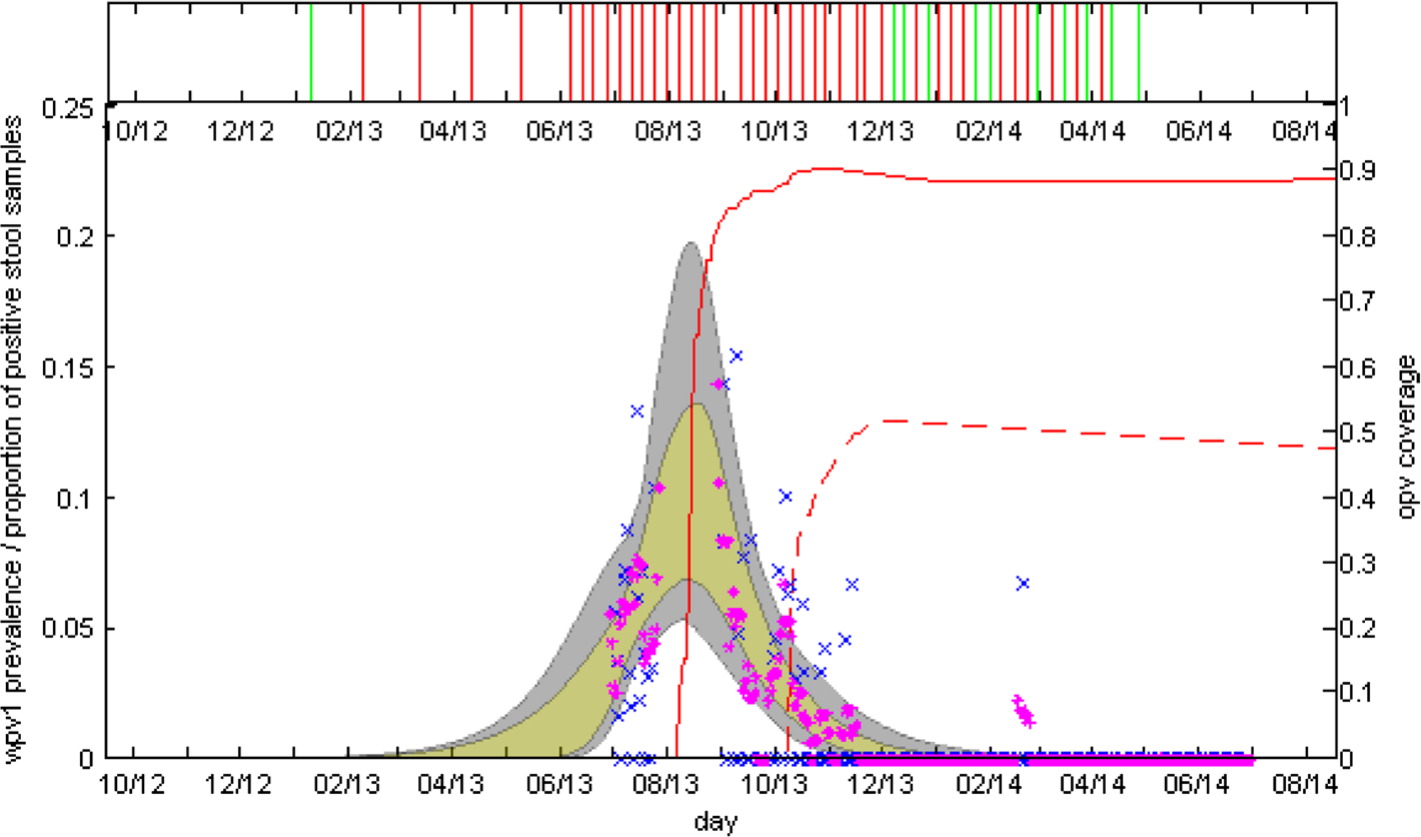
**Top panel:** Results of the ES. Red line indicates a positive finding of WPV1 in one of the four relevant sites (see ‘Methods’). Green line indicates no positive findings. Data shown here is up to the end of April 2014, after which there were no positive findings of WPV1 in any sewage sample. **Bottom panel:** The fit of the model to the stool samples data. The grey area marks the estimated 95 % credible interval of WPV1 prevalence in the modeled population of Bedouin children. The yellow area within the grey area presents a more restricted estimated range of WPV1 prevalence using parameter values whose log-likelihood is within 2 log-likelihood units of the best fit (a commonly used threshold for selection of the more probable fits to the data [34]). The blue x marks the proportion of stool samples positive for WPV1 in each of the days that samples were collected. The magenta dots present a weekly smoothing of the sampled data. For a description of the smoothing and the confidence intervals related to the stochasticity of the observation of the stool data see Figure S7 in Additional file 1. While the likelihood was calculated using the non-smoothed stool sample data, the smoothed data captures the trend of the estimated prevalence better, as it blends in the effect of days with zero positive samples, of which there were many in the second stool survey due to the low number of samples taken each day. The red lines show the cumulative vaccine coverage (right y-axis) of the first (solid line) and second (dashed line) OPV doses in the modeled population

Figure 3 shows the estimated 95 % CI of WPV1 prevalence in the modeled population during the outbreak and its fit to the stool sample data. The figure shows a good fit between the estimated prevalence and the weekly smoothing of the stool data. According to the results of the model fit, the outbreak peaked during the middle of August 2013, immediately after the campaign of the first dose of OPV had begun. However, there is considerable uncertainty regarding the prevalence at the peak of the outbreak, as this is the time between the two stool surveys in which no stool sample data is available. According to the fit, the campaign for the second dose of OPV, which began two months after the first dose campaign, took place when the outbreak was largely over. Together with the low coverage reached in this campaign (max. 53 %) it is therefore no surprise that the model estimates that this campaign had little effect on the outbreak dynamics (with only the first dose of OPV the model projects similar results to those obtained with the two dose campaign (results not shown)).

**Fig. 4:**
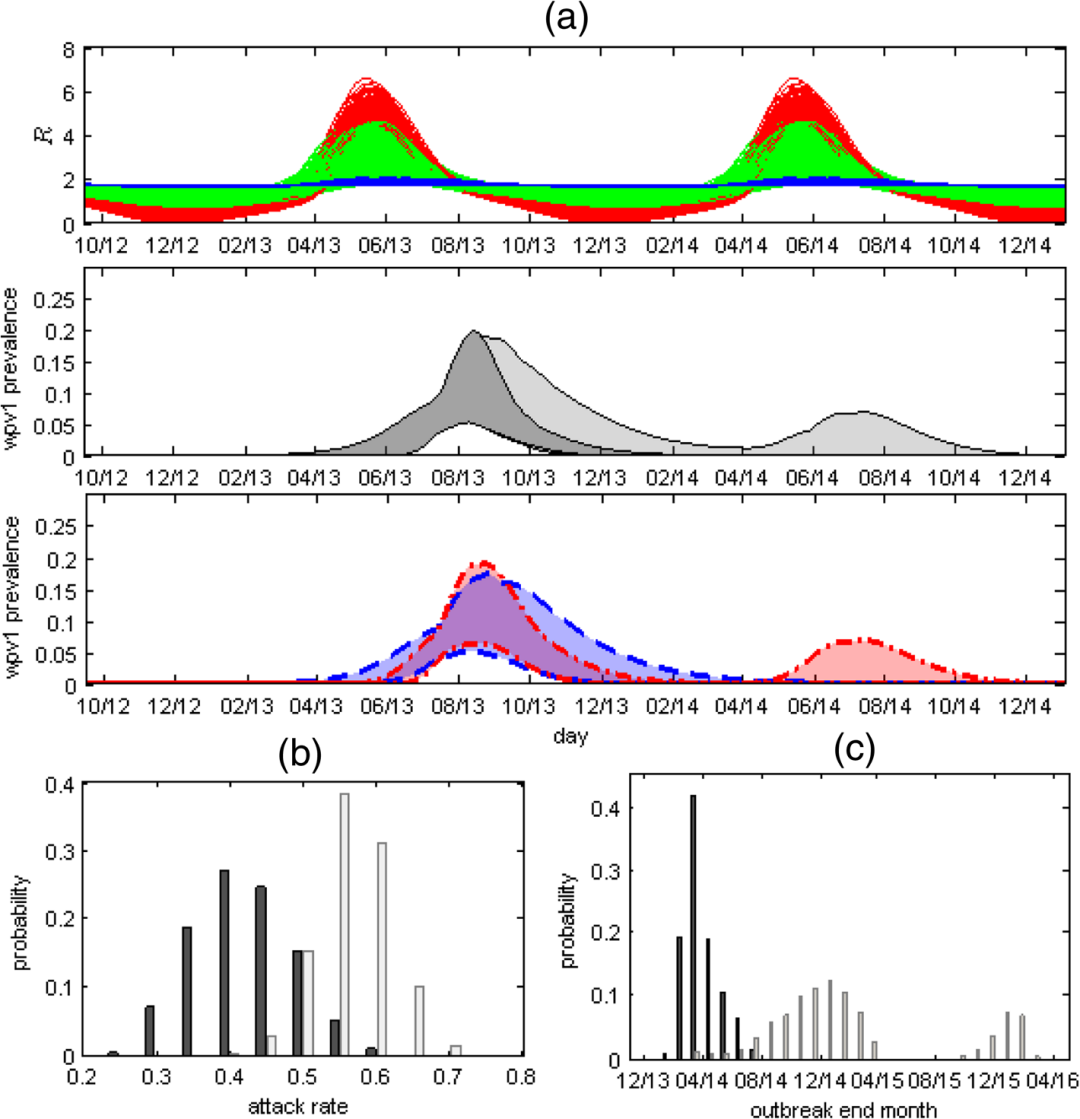
**Top panel:** 1000 plots of the value of the reproductive number (*R*) in time, calculated using Eq. S2 and S4 in Additional file 1 with 1000 values of $\overline{\mathrm{R}}$, *δ* and *ϕ*, randomly sampled out of the values obtained by the MCMC. The range includes plots with no or weak seasonal variation in which $\mathrm{R}=\overline{\mathrm{R}}\sim 1.8$ (blue curves showing results for *δ* ≤ 0.1), plots with strong seasonal variation in which *R* varies from a minimum of close to zero during winter to a maximum of around six during late spring – early summer (red curves showing results for *δ* ≥ 1) and everything in between (green curves). **Middle panel:** 95 % CI of WPV1 prevalence with the oral polio vaccine (OPV) campaign (dark grey) and without the OPV campaign (light grey). **Bottom Panel:** The outcome without the OPV campaign (light grey area in middle panel) depends on the estimated strength of the seasonality. The dashed blue lines depict a subset range of the prevalence without the OPV campaign obtained using weak or no seasonality (*δ* ≤ 0.1), while the red dotted-dashed lines show a subset range of prevalence without the OPV campaign obtained using strong seasonality (*δ* ≥ 1.0). The range obtained using weak seasonality consists of a single long wave, with a tail possibly extending into the first half of 2014, whereas the range obtained using strong seasonality consists of a shorter wave in 2013, with the possibility of a second wave during the second half of 2014. **a** The posterior distribution of the overall attack rate at the end of 2014 with (dark grey bars) and without (light grey bars) the OPV campaign. **b** The posterior distribution for the end time of the outbreak showing the probability of the outbreak ending on a particular month with (dark grey bars) and without (light grey bars) the OPV campaign. With the campaign the model estimates the outbreak ended sometime between January 2014 and October 2014. Without the OPV campaign the model projects the outbreak could have lasted until November 2016 (Table 2)

Figure 4a presents the estimated 95 % CI of WPV1 prevalence with and without the OPV campaign up to the end of 2014 (middle panel). The projections for the prevalence without the OPV campaign were obtained by using the same parameter values given by the MCMC sampling, while removing the OPV vaccinations from the model. Without the vaccination campaign the model projects a possible second epidemic wave taking place during 2014. This second wave is a result of the seasonality in the transmission of WPV1 (Fig. 4a top panel), with the replenishment of the susceptible population due to births a possible contributing factor. In simulations without seasonality there is only a single epidemic but the outbreak is projected to continue for longer than it would have with the OPV campaign (Fig. 4a bottom panel, see also Additional file 1: Figure S8 and Additional file 1: Table S1). The possibility of no seasonality cannot be ruled out from the results of the model fitting, as apparent by the 95 % CI of *δ*, which includes zero (Table 2). In addition, a comparison of the models with seasonality and without seasonality using deviation inference criterion [24] shows no preference for any of the models (Additional file 1: Table S2).

Figures 4b,c show the posterior distributions for the attack rate at the end of 2014 and the end time of the outbreak (set as the time when incidence in the transmission model drops below one infected individual) with and without the OPV campaign (see also Table 2). The mean estimate for the attack rate is 42 % (22–65 %), whereas without the OPV campaign the model projects a mean attack rate of 57 % (35–74 %). The model estimates the end time of the outbreak to be around April 12, 2014 (January 19, 2014, to October 8, 2014). This estimate is in accordance with the results of the ES (Fig. 3 top panel), which is of no surprise as these results were used as part of the inference scheme. When removing the condition related to the results of ES from the inference scheme, the model allows for some scenarios in which a small second epidemic wave occurs during the summer of 2014 (Additional file 1: Figure S10). Without the OPV campaign the model estimates the outbreak could have lasted until November 2016. As a sensitivity test, we recalculated the posterior distribution for the end time of the outbreak when increasing the threshold for extinction to 10 infected individuals (Additional file 1: Figure S11). Using this threshold there is still considerable probability that the outbreak without the OPV campaign would have lasted up to February 2015, but only a very small probability it would have lasted for longer than that. This indicates that stochastic fade-outs of the outbreak during periods of low transmission (which are not accounted for in the deterministic transmission model), are not likely to have prevented the second wave during 2014 but are more likely to have prevented any additional waves after that.

Using the model, we investigated what would have been the effect of different OPV campaigns on the outbreak in terms of the overall incidence and the outbreak duration. We explored the effects of the timing of the vaccination, comparing three alternatives for the start of the campaign: April 5, 2013 – two months after the first indication of WPV1 using ES in retrospect; June 5, 2013 – a week after the outbreak was actually detected; and August 5, 2013 – the time the vaccination campaign actually began. We also explored three possible vaccine coverage scenarios (50 %, 70 % and 90 %) and two dosage scenarios (one dose vs. two doses). The vaccinations were set so that the target coverage was reached within a month of the start date, after which vaccinations were set to maintain the same coverage with the growing population. When modeling the effect of two doses it was assumed the coverage for the second dose is the same as for the first dose and that the second dose is given two weeks after the first dose. For each tested scenario, we ran the model for a period of five years using the 95 % best fitting parameter values given by the MCMC and calculated the mean and 95 % CI of the cumulative incidence and outbreak durations. For this exercise, the end time of the outbreak was set to when the incidence in the transmission model drops below 10 infected individuals, in order to take into account the probability of a stochastic fade-out of the outbreak during the periods of low transmissibility.

**Fig. 5:**
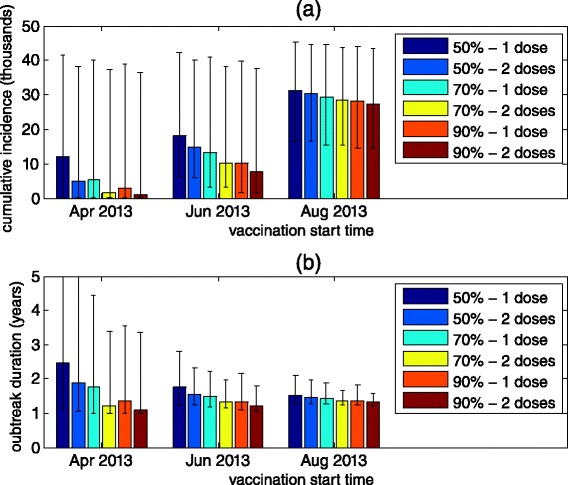
The effect of alternative OPV campaign scenarios on the cumulative incidence of the outbreak (**a**) and the outbreak duration (**b**). The outbreak duration was defined as the time when the incidence in the model drops below 10 infected individuals in order to take into account the probability of a stochastic fade-out of the outbreak during the periods of low transmissibility. Color bars show the mean and error bars show the 95 % CI obtained for each scenario by running the model using the 95 % best fitting parameter values given by the MCMC simulation. The simulations were run for up to five years

Figure 5 summarizes the results of this examination. As expected, the cumulative incidence dropped considerably when the vaccinations were performed earlier in the epidemic (Fig. 5a). A vaccination campaign starting in June would have likely been more effective in reducing the incidence of WPV1 than a campaign starting in August, even if the coverage reached in June would have been just 50 % compared to 90 % in August. This would indicate that starting the vaccination campaign as early as possible should be a primary goal even at the risk of obtaining a reduced coverage. However, the obtained outbreak duration for the different scenarios (Fig. 5b) revealed a conflicting trend. By vaccinating early while not reaching a high enough vaccine coverage, it is possible to actually prolong the outbreak. According to the results shown in Fig. 5b, vaccinating only 50 % of the population with a single dose in June could have resulted in an outbreak lasting for around three years, whereas with vaccination starting in August and reaching 90 % coverage, maximum outbreak duration would be of approximately two years. This effect is even more pronounced had vaccination begun earlier, on April 2013, in which case with coverage of 50 % the epidemic could have lasted for five years and more. The effect is easy to understand in epidemic modeling terms: an early vaccination campaign reaching a coverage that is not high enough to reduce the reproductive number to below unity while leaving a large enough susceptible pool in the population, would lead to a slowly progressing epidemic that would infect only a small proportion of the population but could take a long time to die out (see also Additional file 1: Figure S12). Secondary infections with OPV (which were not accounted for in the model) might reduce this effect to some extent. Early vaccination could result in wider dissemination of OPV as there would be more susceptible individuals in the population. Nevertheless, the increase in OPV coverage through secondary infections should be small due to the reduced transmission capacity of the vaccine virus [21].

With regards to the number of doses, the results presented in Fig. 5 demonstrate that the benefits gained by a second dose of OPV would depend on the timing of the campaign and the coverage reached. When the coverage reached for the first dose is not high enough to mitigate the epidemic, the second dose of OPV could be instrumental in increasing the immunity in the population to the levels needed to stop the epidemic and reducing the risk of a prolonged epidemic. The earlier in the epidemic the vaccination starts, the greater the effectiveness of the second dose of OPV would be. When vaccination starts late in the epidemic, the susceptible pool in the population would already be reduced, so that the coverage needed to mitigate the epidemic would be smaller and the effect of a second dose would also be reduced. With the campaign starting at the actual time it did (August 2013), the model indicates that the effect of the second dose of OPV would have been limited, even under a more optimal scenario in which anyone that receives a first dose receives a second dose two weeks later.

## Discussion

In this work, an epidemic transmission model was fitted to the unique stool sample data collected during the reintroduction of poliovirus to Israel. The inclusion of a condition set according to information gathered by ES helped to reduce uncertainty in the results of the model fitting (Additional file 1: Figure S9–S10 and Additional file 1: Table S3). To our knowledge, this is the first attempt at estimating the dynamics of a silent transmission of poliovirus using such data. Using MCMC we obtained the posterior distributions for the model parameters (Fig. 2 and Table 2). The results of the model fitting suggest the outbreak had peaked during August 2013, after the initiation of the first dose of the OPV campaign, and had ended around April 2014 (Fig. 3 and Table 2). The results also indicate that the OPV vaccination campaign was most likely effective in curtailing the WPV1 transmission, both reducing the overall attack rate and shortening the duration of the outbreak (Fig. 4 and Table 2).

Our estimates for the reproductive number with maximum seasonal variation range between zero and six (Fig. 4a, top panel). As we model the transmission of WPV1 within an IPV vaccinated population, these values might not be similar to the values of the reproductive number in a completely susceptible population (known as the basic reproductive number – *R*_0_). The relationship between the reproductive number in our settings and the reproductive number in a naïve population depends on the protective effect of IPV in terms of its ability to reduce poliovirus transmission. One such suggested protective effect of IPV is shortening of the infectious period [25–27]. Our estimates of the mean infectious period in the range of 8–25 days are considerably shorter than the estimated range of 28–49 days in naïve populations [28–30], which may serve as support for such claims. If IPV does shorten the period of infection, then the value of the reproductive number in a naïve population should be higher than our estimates, as the reproductive number is proportional to the mean infectious period (Additional file 1: Eq. S2).

The modeling suggests that the late initiation of the OPV campaign reduced its effectiveness (Fig. 5). The two months between the first identification of the WPV1 using ES and the initiation of the OPV campaign were used by the Israeli Ministry of Health to validate the likely prevalence of infection and determine the epicenter of transmission (through the first stool survey), and to prepare for the campaign, which included the acquisition of the bOPV stockpiles and the organization of a communication effort, targeting both physicians and the general public, in order to increase the public trust in the vaccination campaign and achieve higher vaccination coverage [10]. Balancing speed of response against achieving high coverage is a dilemma that decision makers face in the event of a polio outbreak. Moreover, we show that the effectiveness of the outbreak control measures can be assessed using different criteria, namely overall incidence or outbreak duration, which adds another layer of complexity. An early vaccination campaign with a low to medium coverage could have significantly reduced the incidence of WPV1 at the cost of possibly prolonging the outbreak by a significant measure (Fig. 5). The duration of an outbreak is especially important when considering global polio eradication efforts. In a long outbreak, the probability of exporting cases to other locations can increase. In addition, a country faces the risk of being labeled a re-established transmission country if there is evidence for WPV transmission over a year, despite no paralytic cases.

In Israel, decision-makers also faced a second dilemma of whether to use a single or two doses of OPV in the campaign. A multi-dose vaccination campaign has several drawbacks compared to a single dose campaign. The main concern in Israel was that multi-dose vaccination will achieve lower public acceptance compared to a single dose campaign, which would lead to lower overall vaccine coverage (in fact, the coverage of the second OPV dose reached only 53 %). In addition, a multi-dose campaign would have further exhausted the public health resources necessary for maintaining the routine vaccination schedule in Israel. With the 90 % coverage achieved with the first dose of OPV, the model showed that the second dose had almost no impact on transmission. Even under an optimal outbreak response scenario (two doses given two weeks apart starting as soon as transmission was detected) the second dose would have had limited impact (Fig. 5). A second dose would have had bigger impact if vaccination had begun even earlier (assuming earlier detection) and if the first dose coverage had been lower. Obviously, the importance of a second dose (and third and fourth doses) is greater in populations in which the efficacy of OPV is low, since in these populations even a high coverage of a single dose of OPV might not be enough to reach the levels of protection necessary to prevent an epidemic. Given the challenges in achieving high coverage in some other newly infected countries, multi-dose campaigns, which also offer additional opportunities for vaccinating children who missed the first dose, are more beneficial and make an important contribution to global polio eradication efforts.

Our model focuses on the transmission among children under the age of 10, the birth cohorts that received only IPV as part of their routine vaccination schedule and in whom all the positive stool samples were found. While there may have been some transmission in older OPV-vaccinated age groups in whom mucosal immunity had waned, there was no indication for that in the stool samples gathered in the Bedouin population (no positives among 203 stool samples from ≥10 year olds – see Additional file 1: Figure S1). We acknowledge the possibility that older children and adults have a potential to play a role in the transmission of the disease. The model also does not take into account any spatial dynamics for which spread between disparate communities by adults may play a role. As in any study attempting to make inference by fitting a model to data, there was a need to balance model complexity with the ability to estimate the model parameters, which is limited by the availability and the quantity and quality of the data. Unfortunately, this limited our ability to investigate important issues such as waning immunity in the older age groups and their potential role in the spatial and temporal spread of the epidemic. It would be interesting to consider more complex models in future theoretical modeling studies or in any future outbreaks that generate more detailed age and geographically stratified stool sample data.

## Conclusions

The Israeli experience illustrates the importance of OPV in interrupting WPV transmission, even in a developed country setting with a very high coverage of IPV. While the larger family size and more crowed living conditions in the Bedouin community may have facilitated WPV transmission in this population, there was no evidence of transmission despite importations into the West Bank and Gaza, where conditions are similar but a sequential IPV/OPV schedule is used [31]. The fact that transmission within the Bedouin population of southern Israel was mostly limited to children below 10 (i.e., individuals vaccinated with IPV only) further illustrates the consequences of the loss of the mucosal immunity induced by OPV. Following the switch from tOPV to bOPV in April 2016 [21], the population immunity to type 2 poliovirus will, in time, drop below the required herd immunity level and, in such a situation, reintroduction of WPV2, or the more likely scenario of emergence of a circulating vaccine derived strain, can initiate an epidemic. The WHO Strategic Advisory Group of Experts has recommended that the outbreak response in such an event requires the use of monovalent OPV type 2 to generate mucosal immunity and interrupt transmission [32]. Our experience and modeling provides evidence in support of this view and can be used to develop optimal vaccination policies in case of future reintroductions of the virus.

The Israeli experience also highlights the importance of environmental surveillance for the early detection of poliovirus transmission, especially in countries with high IPV vaccine coverage. In Israel, had there only been acute flaccid paralysis (AFP) surveillance, the probability of detecting the outbreak early would have been low and would only have occurred after the devastating consequence of a paralytic case; transmission could have gone unnoticed in the absence of a paralytic case or with imperfect AFP surveillance. Early detection of poliovirus transmission using ES is now an essential component of the global polio eradication strategy, which, in 2016, entered a new phase with the withdrawal of OPV2 [33]. The outbreak in Israel demonstrates that the introduction of IPV to mitigate the consequences of an emergence of a circulating vaccine-derived poliovirus will have a limited effect on transmission by itself. However, establishing ES will allow early initiation of outbreak response measures which, combined with the protection afforded by IPV against paralysis, will reduce the burden of AFP.

## Abbreviations

AFP, acute flaccid paralysis; bOPV, bivalent oral polio vaccine; CI, credible intervals; ES, environmental surveillance; IPV, inactivated polio vaccine; MCMC, Markov chain Monte Carlo; OPV, oral polio vaccine; WPV, wild poliovirus; WPV1, wild poliovirus type 1.

## Additional files

Additional file 1: Supporting Information. (PDF 912 kb) Additional file 2: Stool Sample Data. (CSV 4 kb)
