# Erratum to: Modeling the spread of polio in an IPV-vaccinated population: lessons learned from the 2013 silent outbreak in southern Israel

**DOI:** 10.1186/s12916-016-0666-7

**Published:** 2016-08-19

**Authors:** Rami Yaari, Ehud Kaliner, Itamar Grotto, Guy Katriel, Jacob Moran-Gilad, Danit Sofer, Ella Mendelson, Elizabeth Miller, Amit Huppert, for the POG group

**Affiliations:** 1 Bio-statistical Unit, The Gertner Institute for Epidemiology and Health Policy Research, Chaim Sheba Medical Center, Tel Hashomer, 52621 Israel; 2 Biomathematics Unit, Department of Zoology, Faculty of Life Sciences, Tel Aviv University, 69978 Tel Aviv, Israel; 3 Public Health Services, Ministry of Health, Jerusalem, Israel; 4 Faculty for Health Sciences, Ben-Gurion University of the Negev, Beer-Sheva, Israel; 5 Department of Mathematics, ORT Braude College, Karmiel, Israel; 6 Central Virology Laboratory, Ministry of Health, Chaim Sheba Medical Center, Tel Hashomer, Israel; 7 School of Public Health, the Sackler Faculty of Medicine, Tel Aviv University, Tel Aviv, Israel; 8 Public Health England Immunisation, Hepatitis and Blood Safety Department, 61, Colindale Avenue, London, UK

## Erratum

After publication of the original article [1], it came to the authors’ attention that there was an labelling error contained within the caption of Fig. 4. The **a, b, c** labelling in the caption did not correctly correspond to the panels within the Figure itself. The article has been updated to rectify this error, and Fig. 4 is published with the correct caption in the erratum.

**Fig. 4:**
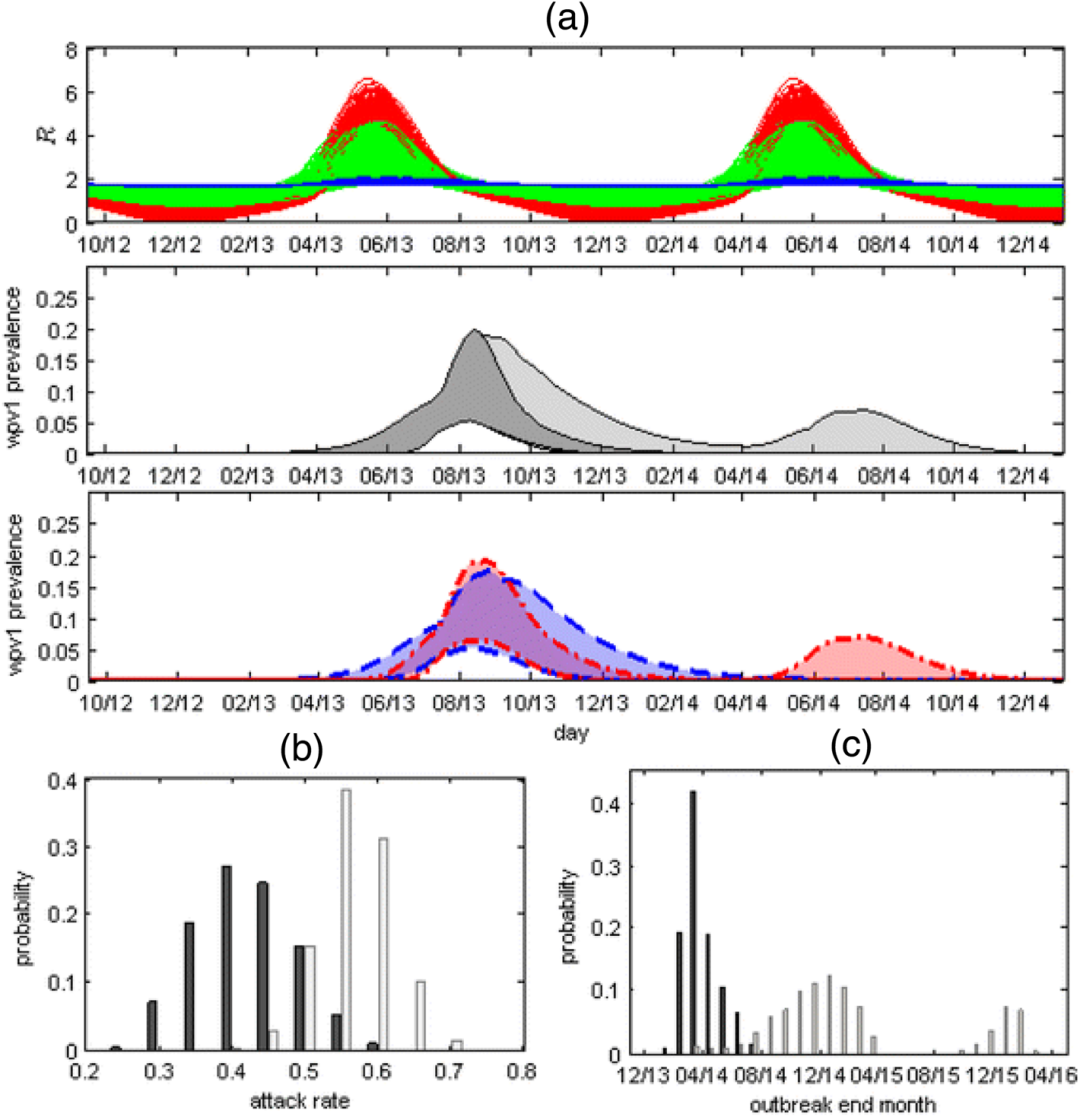
**a** Top panel: 1000 plots of the value of the reproductive number (*R*) in time, calculated using Eq. S2 and S4 in Additional file 1 with 1000 values of $\overline{\mathrm{R}}$, *δ* and *ϕ*, randomly sampled out of the values obtained by the MCMC. The range includes plots with no or weak seasonal variation in which $\mathrm{R}=\overline{\mathrm{R}}\sim 1.8$ (blue curves showing results for *δ* ≤ 0.1), plots with strong seasonal variation in which R varies from a minimum of close to zero during winter to a maximum of around six during late spring – early summer (red curves showing results for *δ* ≥ 1) and everything in between (green curves). Middle panel: 95 % CI of WPV1 prevalence with the oral polio vaccine (OPV) campaign (dark grey) and without the OPV campaign (light grey). Bottom Panel: The outcome without the OPV campaign (light grey area in middle panel) depends on the estimated strength of the seasonality. The dashed blue lines depict a subset range of the prevalence without the OPV campaign obtained using weak or no seasonality (*δ* ≤ 0.1), while the red dotted-dashed lines show a subset range of prevalence without the OPV campaign obtained using strong seasonality (*δ* ≥ 1.0). The range obtained using weak seasonality consists of a single long wave, with a tail possibly extending into the first half of 2014, whereas the range obtained using strong seasonality consists of a shorter wave in 2013, with the possibility of a second wave during the second half of 2014. **b** The posterior distribution of the overall attack rate at the end of 2014 with (dark grey bars) and without (light grey bars) the OPV campaign. **c** The posterior distribution for the end time of the outbreak showing the probability of the outbreak ending on a particular month with (dark grey bars) and without (light grey bars) the OPV campaign. With the campaign the model estimates the outbreak ended sometime between January 2014 and October 2014. Without the OPV campaign the model projects the outbreak could have lasted until November 2016 (Table 2)
