# Supplementary material for: Modeling the spread of polio in an IPV-vaccinated population: lessons learned from the 2013 silent outbreak in southern Israel
Source: BMC Med. 2016 Jun 23;14:95. doi: 10.1186/s12916-016-0637-z (PMC4918056; doi:10.1186/s12916-016-0637-z)
Supplement: Additional file 1: — Supporting Information. (PDF 912 kb) [file 12916_2016_637_MOESM1_ESM.pdf]

Modeling the spread of polio in an IPV-vaccinated population; lessons learned from the 2013 silent outbreak in southern Israel

Supplementary Information

Rami Yaari[1,2], Ehud Kaliner[3], Itamar Grotto[3,4,] Guy Katriel [5],Jacob Moran-Gilad[3,4], Danit Sofer[6], Ella Mendelson[6,7], Elizabeth Miller[8] and Amit Huppert[1,7] for the POG[9] group.

1) Bio-statistical Unit, The Gertner Institute for Epidemiology and Health Policy Research, Chaim Sheba Medical Center, Tel Hashomer, 52621 Israel

2) Biomathematics Unit, Department of Zoology, Faculty of Life Sciences, Tel Aviv University, 69978 Tel Aviv, Israel

3) Public Health Services, Ministry of Health, Jerusalem, Israel

4) Faculty for Health Sciences, Ben-Gurion University of the Negev, Beer-Sheva, Israel

5) Department of Mathematics, ORT Braude College, Karmiel, Israel

6) Central Virology Laboratory, Ministry of Health, Chaim Sheba Medical Center, Tel-Hashomer.

7) School of Public Health, the Sackler Faculty of Medicine, Tel-Aviv University

8) Public Health England Immunisation, Hepatitis and Blood Safety Department, 61, Colindale Avenue, London, United Kingdom

9) The POG (Polio Outbreak Group), by alphabetical order of and within Institutes: Anis E[3], Kopel E[3], Manor Y[6], Mor O[6], Shulman L[6], Singer SR[3], Weil M[6]

1. Results of stool survey by age

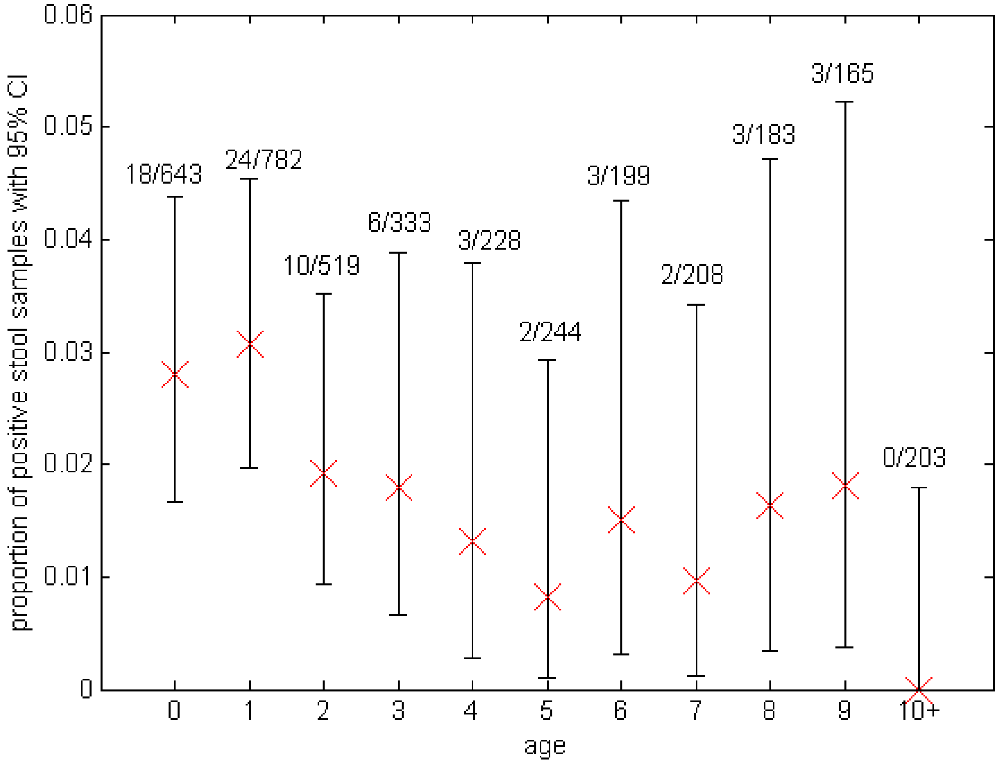

Figure S1: The results of the stool surveys (after removing of repeat samples – see data section in the main text) conducted in the Bedouin population of southern Israel between July 2013 and June 2014. 'x' marks the proportion of positive tests in each age-group and the bars mark the 95% confidence intervals given by the binomial distribution using Clopper-Pearson. The exact numbers of positive and total tests are also given in the figure. Note that above the age of ten no positive samples were found among 203 sampled individuals - similar to the number of individuals sampled in each of the one year age-groups 5, 6, 7, 8 and 9, in which positive samples were found. This is an indication that individuals above 10, who were vaccinated with OPV as part of their routine vaccination schedule, played a negligible role in transmission of WPV1.

2. Transmission model

Following is the formulation of the deterministic, discrete-time SEIR transmission model that is described schematically in figure 1 of the main text:

(S1a) $$S_1(t) = S_1(t-1) - \beta(t) \cdot I(t-1) \cdot \frac{S_1(t-1)}{N(t-1)} - h_1(t) \cdot v_{OPV1}(t) + b(t)$$

(S1b) $$S_2(t) = S_2(t-1) - \beta(t) \cdot I(t-1) \cdot \frac{S_2(t-1)}{N(t-1)} + (1-\rho) \cdot h_1(t) \cdot v_{OPV1}(t) - h_2(t) \cdot v_{OPV2}(t)$$

(S1c) $$S_3(t) = S_3(t-1) - \beta(t) \cdot I(t-1) \cdot \frac{S_3(t-1)}{N(t-1)}` + (1-\rho) \cdot h_2(t) \cdot v_{OPV2}(t)$$

(S1d) $$E(t) = E(t-1) + \beta(t) \cdot I(t-1) \cdot \frac{[S_1(t-1) + S_2(t-1) + S_3(t-1)]}{N(t-1)} - 1/d_L \cdot E(t-1)$$

(S1e) $$I(t) = I(t-1) + 1/d_L \cdot E(t-1) - \gamma \cdot I(t-1)$$

(S1f) $$R(t) = R(t-1) + 1/d_I \cdot I(t-1) + \rho \cdot \left(h_1(t) \cdot v_{OPV1}(t) + h_2(t) \cdot v_{OPV2}(t)\right)$$

(S1g) $$N(t) = N(t-1) + b(t)$$

S_1, S_2 and S_3 denote groups of individuals susceptible to infection with polio. S_1 denotes individuals that were not yet vaccinated with OPV, S_2 denotes individuals that received one dose of OPV but have not gained mucosal immunity and S_3 denotes individuals that received two doses of OPV but have not gained immunity. E denotes exposed individuals who are infected but not yet infectious, I denotes infectious individuals and R denotes recovered or immune individuals who gained their immunity via infection with the wild poliovirus or via vaccination with OPV. $N(t)$ denotes the total population size on day t, which grows by $b(t)$ newborn susceptible individuals. We set $b(t) = \mathrm{B} = 21 \approx 0.035 * 220{,}000/365$, assuming a fixed number of daily newborns and based on a 3.5% yearly birth rate in the Bedouin population of southern Israel, which reached 220,000 in 2013 (see also fig. S2). The fixed number of daily newborns is a good approximation for the 2-3 years period in which the model is run. The model assumes homogeneous mixing of the population. The number of susceptible individuals of group $\mathrm{S_X}$ that become infected on day t depends on the transmission rate on day t - $\beta(t)$, the number of infectious individuals and the proportion of susceptible individuals of groups $\mathrm{S_X}$ in the population on the previous day. The transmission rate on day t is given by the formula:

(S2) $$\beta(\mathrm{t}) = \overline{\mathrm{R}} \cdot \big(1 + \omega(t)\big)/d_I$$

where $\mathrm{d_I}$ is the mean infectious period, $\overline{\mathrm{R}}$ is the mean reproductive number and $\omega(t)$ is a periodic function with the property $E\big(\omega(t)\big) = 0$ describing the seasonal variation in the reproductive number, which is modeled using the estimated seasonality in the southern US states during the pre-vaccination era (see 'Modeling Seasonality' section below).

Exposed individuals become infectious at rate $1/d_L$ (d_L being the mean period of latency) and infectious individuals recover at rate $1/d_I$. $v_{OPV1}(t)$ and $v_{OPV2}(t)$ denote the number of individuals vaccinated with a first and second dose of OPV on day t. We used the data provided by the Israeli Ministry of Health, with a delay of one week, as this is the time it takes for the vaccines to build up protective immunity [1]. ρ denotes the per-dose efficacy of OPV (i.e., the probability that the vaccine will build up protective intestinal immunity, as in [2–4]]). Finally, $h_1(t)$ and $h_2(t)$ denote the probability that a first and second dose of OPV given on day t, will be given to susceptible individual. These probabilities are calculated according to the fraction of susceptible individuals in the target population for each dose (since the first dose was only given to those who did not receive one yet and the second dose was given only to individuals who received a first dose):

(S3a) $$h_1(t) = \frac{S_1(t-1)}{\left(N(t-1) - \sum_{\dot{t}=1}^{t-1} v_{OPV1}(\dot{t})\right)}$$

(S3b) $$h_2(t) = \frac{S_2(t-1)}{\sum_{\dot{t}=1}^{t-1} v_{OPV1}(\dot{t}) - \sum_{\dot{t}=1}^{t-1} v_{OPV2}(\dot{t})}$$

The initial conditions of the model for the start of the epidemic are $I(t_0) = 1$, $S_1(t_0) = N(t_0) - 1$ and $S_2(t_0) = S_3(t_0) = E(t_0) = R(t_0) = 0$, where t_0 defines the day in which the first infection in Israel occurred (t_0=1 was set to September 15, 2012 – see below). For $t < t_0$ we set $I(t) = 0$.

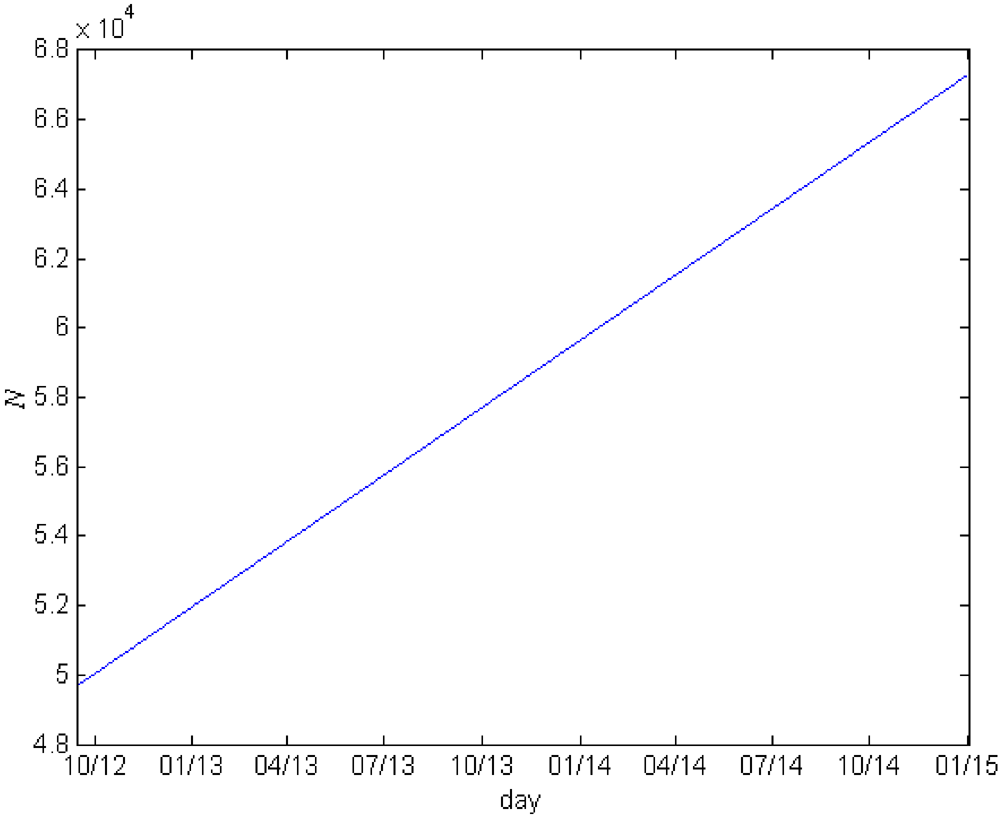

Figure S2: Size of the modeled population in time. The initial population size on September 15, 2012 was calculated as $N(1) = 57{,}882 - 21 * 390 = 49692$, where $57{,}882$ is the number of individuals under ten in the Bedouin population who were being targeted for vaccination with OPV by October 10, 2013, and $21 \approx 0.035 * 220{,}000/365$ is the estimated number of daily newborns based on a 3.5% yearly birth rate in the Bedouin population of southern Israel, which reached 220,000 in 2013.

3. Modeling seasonality

In order to model the seasonal variation in the reproductive number we employ information available from a study modeling the spread of WPV in the US in the 1930s-1950s [5]. The study estimated the seasonality of WPV in the different US states. We used the estimated seasonality for ten southern US states (Alabama, Arizona, California, Florida, Georgia, Louisiana, Mississippi, New Mexico, South Carolina and Texas) that share the same latitudes with Israel (30N-33N), as the study indicated that the timing of seasonality of WPV has a latitudinal gradient. Using these data we modeled the seasonal variation $\omega(t)$ (appearing in eq. S2) as:

(S4) $$\omega(t) = \delta \cdot \overline{\omega}(\mathrm{mod}(\mathrm{t} + (\overline{\phi} - \phi), 365))$$

where $\overline{\omega}(\mathrm{t})$, $1 \leq \mathrm{t} \leq 365$, is a function of the mean seasonality of the ten US states obtained by interpolating the monthly estimates into daily data, centering the seasonal functions so that their peaks align at the day of the mean peak time, taking the mean of the functions and normalizing it (dividing by the mean and subtracting by one) so that $E\left(\overline{\omega}(\mathrm{t})\right) = 0$ (fig. S3a-d). The parameters δ and Φ are model parameters that allow to control the amplitude and peak time of the seasonal variation. δ sets the amplitude so that $\delta = 0$ means there is no seasonal variation while $\delta = 1$ means a seasonal variation similar to the mean seasonal variation of $\overline{\omega}$. ϕ sets the peak time so that when $\phi = \overline{\phi} \equiv argmax(\overline{\omega}) = 156$ (i.e., June 5) the same peak time as is in $\overline{\omega}$ is obtained. Using the variance in the amplitude and peak time of the seasonal variation estimated for the ten southern US states we obtain prior distributions for ϕ and δ for our Bayesian inference (fig. S3e-f). The prior distribution for ϕ was set as $N(156{,}17.55)$ evaluated at the range [1,365] (with steps of 1 day), according to the mean and standard deviation of the peak time in the seasonal curves shown in fig. S3b. The prior distribution for δ was set as $N(1{,}0.414)$ evaluated at the range [0,3] (with steps of 0.01), according to the mean and standard deviation obtained for the ratios of the amplitudes of the normalized seasonal curves for each of the US states and the amplitude of the normalized mean seasonal curve $\overline{\omega}$ (curves shown in fig. S3d).

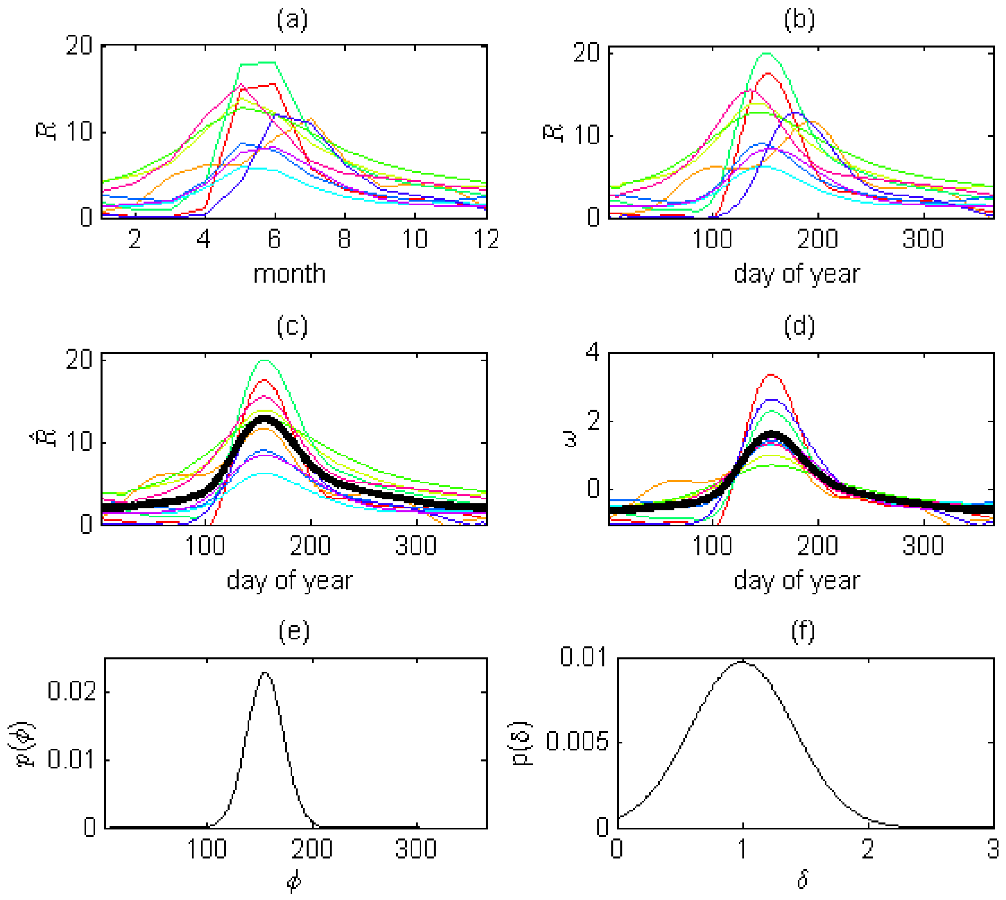

Figure S3: Modeling the seasonal variation of the reproductive number of WPV:

(a) Estimates of the monthly variation in the reproductive number of WPV in ten southern US states obtained by fitting epidemic data from the 1930s-1950s (data taken from [5]).

(b) The curves from (a) after interpolation into daily data.

(c) The curves from (b) after centering them so that their peaks align at the mean peak time. The thick black curve is the mean of the ten seasonal curves.

(d) The curves from (c) after normalizing each of the curves so that the mean of each curve is zero. The thick black curve is the normalized mean seasonality ($\bar{\omega}$ in eq. S4).

(e) Prior distribution for the peak time of the seasonal variation (ϕ) obtained using a normal distribution $N(156,17.55)$, set with the mean and standard deviation of the peak times of the curves in (b).

(f) Prior distribution for the amplitude of the seasonal variation (δ) obtained using a normal distribution $N(1,0.414)$, set with the mean and standard deviation of the amplitude ratios between the normalized curves for each state and the mean normalized curve given in (d).

4. Observation model

We used a binomial observation model to link the prevalence given by the transmission model to the stool survey data:

(S5)

$$T^{+}(t) \sim Binomial\left(T(t), \frac{I(t)}{N(t)}\right)$$

where $T(t)$ is the number of total samples taken on day t, $T^{+}(t)$ is the number of positive samples found on day t (see supplementary data file) and $I(t)/N(t)$ is the prevalence of polio in the population on day t according to the transmission model. The likelihood function for the model is the probability of obtaining the stool survey data given the model and a set of values for the model parameters. Denoting $p_B(t) = I(t)/N(\mathrm{t})$ and $\theta = \{d_I, \overline{\mathrm{R}}, \delta, \phi, \rho, \mathrm{t}_0\}$ this function can be written as:

(S6)

$$\mathrm{L}(\theta) = \prod_{\mathrm{t}=1}^{\mathrm{t_{end}}} \binom{\mathrm{T(t)}}{\mathrm{T^{+}(t)}} \cdot \mathrm{p_B(t)}^{\mathrm{T^{+}(t)}} \cdot \left(1 - \mathrm{p_B(t)}\right)^{\mathrm{T(t)} - \mathrm{T^{+}(t)}}$$

In addition, as mentioned in the main text, we employed the information available from ES by enforcing a limitation on the likelihood function so that if a set of parameter values θ does not maintain the following condition:

(S7)

$$\frac{I(t_D)}{N(t_D)} > \frac{I(\acute{t})}{N(\acute{t})} \quad \text{for each } \acute{t} > t_{ND}$$

where t_D is the time of the initial detection of WPV1 in the sewage (Feb. 6, 2013) and t_{ND} is the time two months after the last detection of WPV1 in the sewage (June 3, 2014), then we set $\mathrm{L}(\theta) = 0$. The logic behind this limitation is that according to ES, the prevalence of WPV1 after June 3, 2014, when it was no longer detected in the sewage, should not have been higher than the prevalence at the time when WPV1 became detectable in the sewage on February 6, 2013, since otherwise it should have been detected by the ES.

5. Bayesian inference

We adopted a non-informative uniform prior for the for the duration of infectiousness (d_I) in the range [7,49] days based on estimates ranging from 4 to 7 weeks in naïve populations [6–8], and the suggestions that the period of infectiousness can be shorter in a population vaccinated with IPV [9–11]. For the mean reproductive number ($\bar{R}$) we set a uniform prior in the range [1,10], as 1 is the threshold value above which an epidemic can occur, and 10 is the high end estimate for $\bar{R}$, attributed to a situation in a country with poor sanitary conditions [12]. For the two seasonality parameters (δ and ϕ) we employed prior distributions obtained from estimates of the seasonal variation of WPV in southern US states, as described above. The prior distribution for the per-dose efficacy of OPV was set as $N(0.56,0.23)$ according to estimates given in a recent meta-analysis study [13]. We set a uniform prior for the initiation time of the epidemic (t_0) in the range [1,145] (which implies initiation dates between September 15, 2012 and February 6, 2013). This lower limit of this range was set based on the results of a phylogenetic study, analyzing poliovirus isolates from Israel and Egypt, that indicated the Israeli and Egyptian WPV1 lineages have diverged around mid-September 2012 [14]. The upper limit for this range was set according to the timing of the first identification of WPV1 in Israel using ES [15].

We explored the posterior distribution of the model parameters θ by employing Markov Chain Monte Carlo (MCMC) sampling performed using the slice sampling method [16] (implemented using the slicesample function in matlab) on the sum of log $L(\theta)$ given in eq. S6 and the log of the prior distributions, with the additional limitation given in eq. S7. We discarded the first 10,000 iterations as a burn-in period and used a thinning of 1 in 10 samples as to avoid auto-correlation between adjacent samples. The remaining 10,000 samples after the burn-in period and the thinning were used to obtain the posterior distribution for the model parameters. 95% credible intervals were obtained by removing the 5% of the samples with the lowest likelihood and computing the range within the remaining samples. Figure S4 shows the traces of the parameter values obtained by the MCMC. Figure S5 shows pairwise scatter plots of the parameter values obtained by the MCMC and figure S6 shows scatter plots depicting the relationship between the parameter estimates and the obtained results in terms of the overall attack rate by the end of 2014 and the duration of the outbreak. Positive correlations were obtained between the mean reproductive number ($\bar{R}$) and the duration of infection (d_I) and

between $\bar{R}$ and the efficacy of OPV (ρ) (fig. S5). We also found a strong negative correlation between d_I and the overall attack rate (fig. S6). Since we fit prevalence data, a longer period of infection is able to help fit the same data with fewer cases, thus explaining the negative correlation between $\mathrm{d_I}$ and the attack rate. For this reason we also obtain smaller reproductive number values for larger values of $\mathrm{d_I}$. A larger ρ necessitates a larger $\bar{R}$ to obtain the same number of cases, which explains the positive correlation between these two parameters.

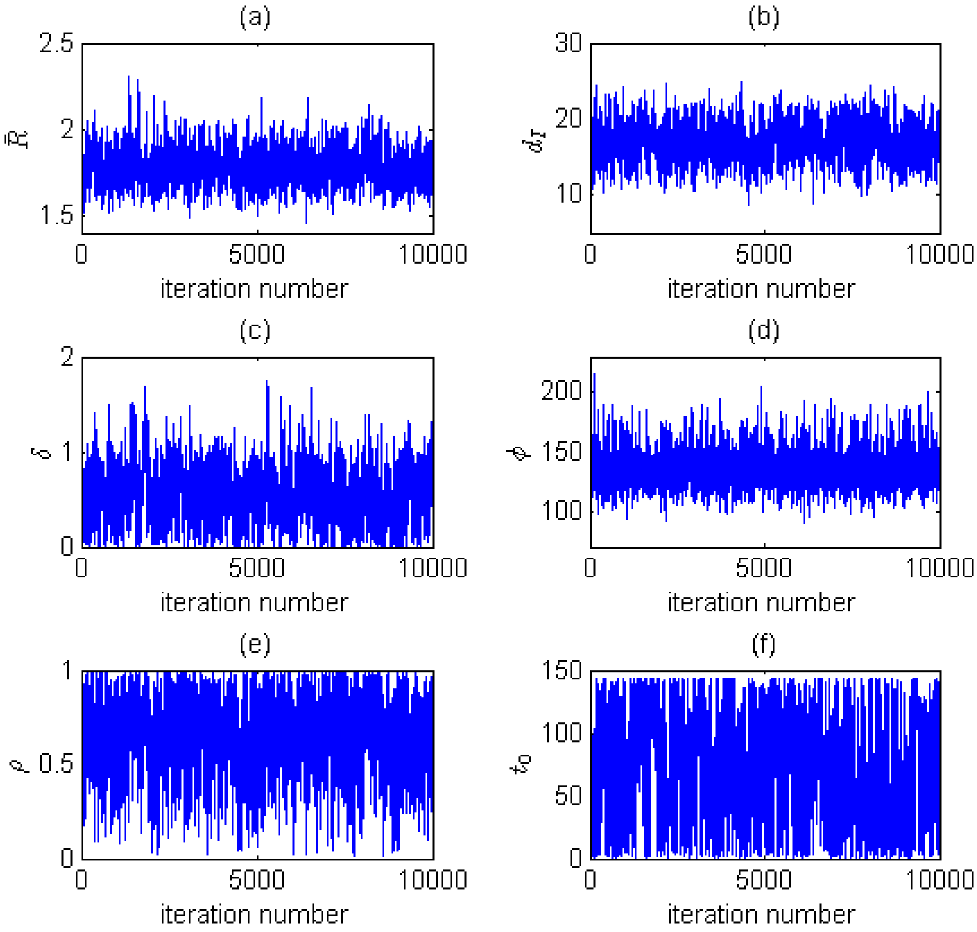

Figure S4: Traces of the model parameter values as obtained by the MCMC sampling.

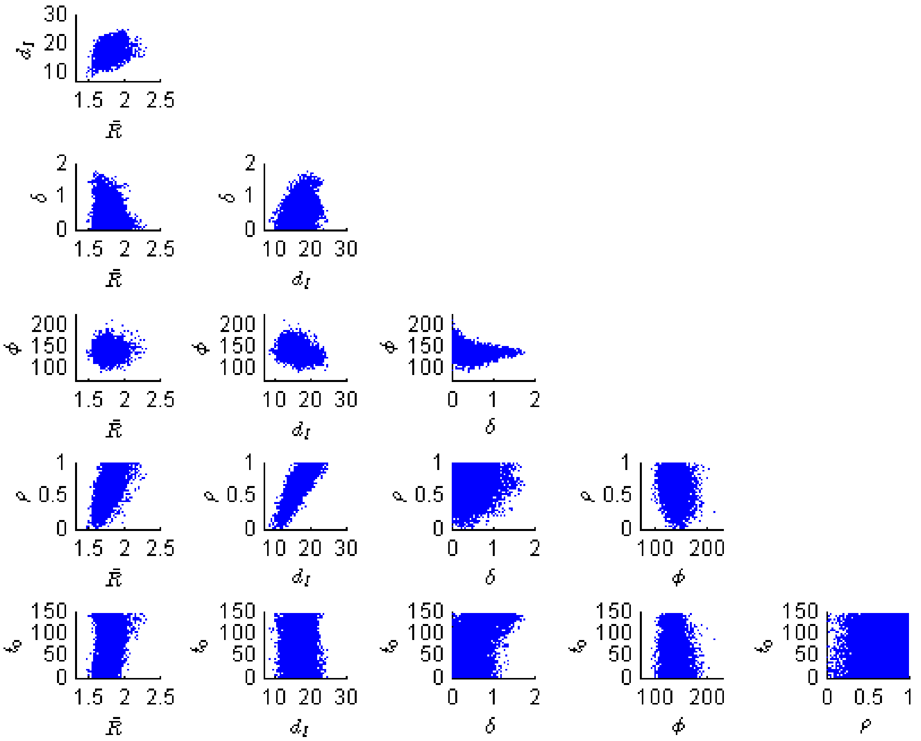

Figure S5: Pairwise scatter plots of the model parameters, using the 95% best fitting samples obtained by the MCMC.

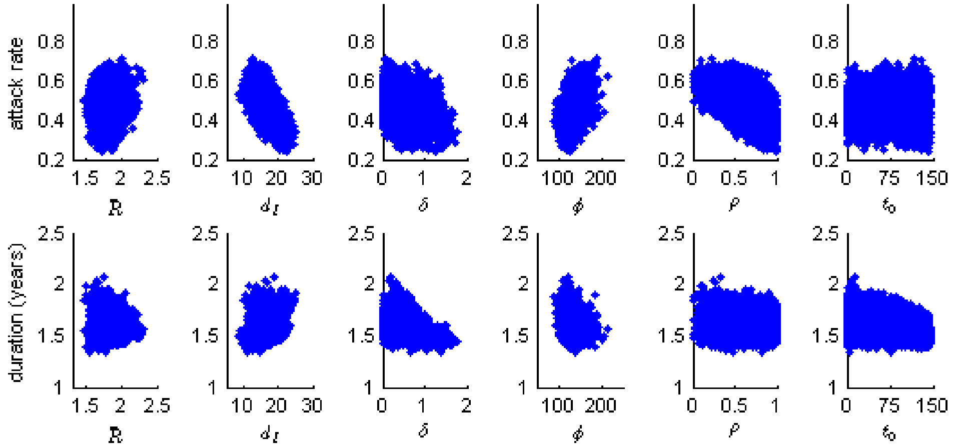

Figure S6: Scatter plots of the 95% best fitting model parameters values obtained by the MCMC and the corresponding outcomes of the epidemic (attack rate at the end of 2014 and the epidemic duration given in years).

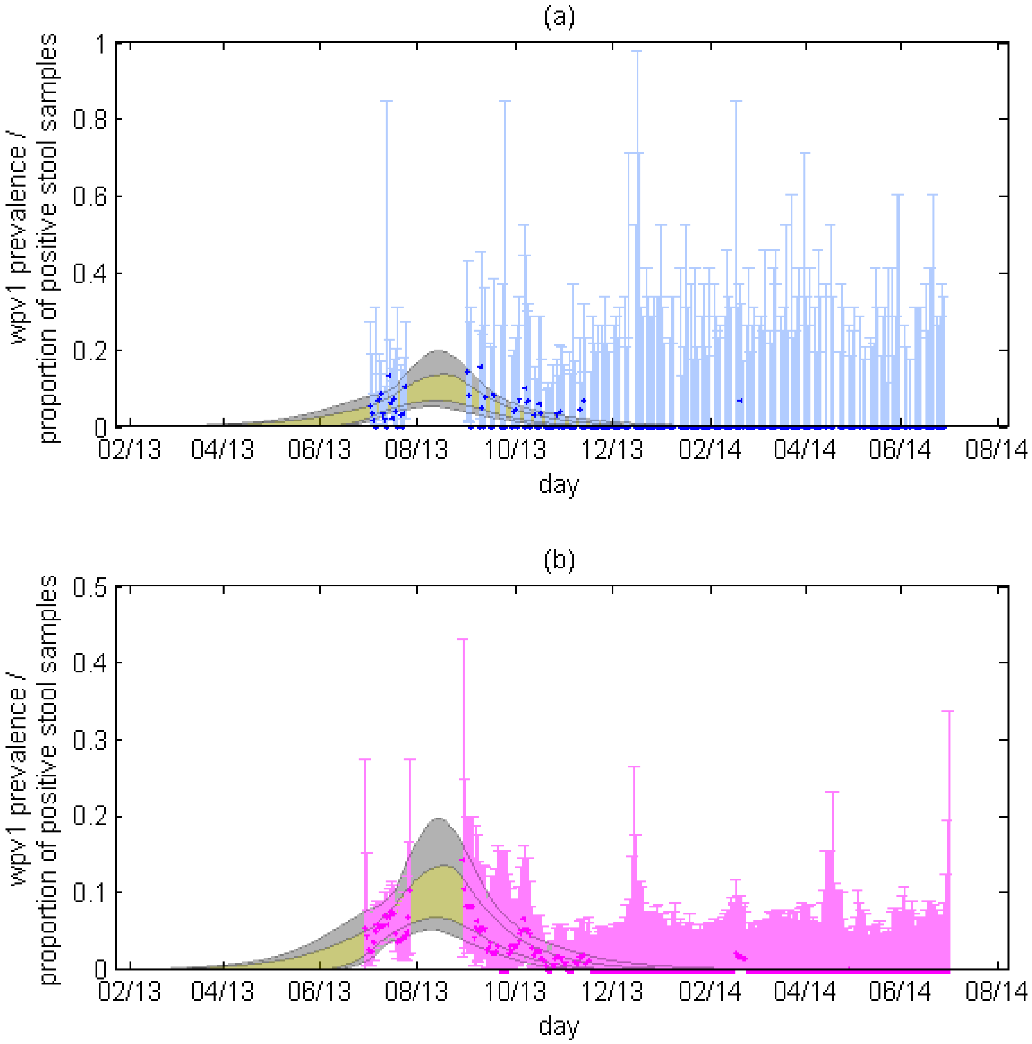

Figure S7: The grey and yellow areas show the 95% credible intervals for WPV1 prevalence and the range given by parameter values whose log-likelihood is within 2 log-likelihood units of the best fit obtained by the model fitting respectively (as in fig. 3 of the main text). The markers and error bars show the proportions of positive stool samples and the related 95% confidence intervals (CI) given by the binomial distribution using Clopper-Pearson.

(a) The proportions and 95% CI of the daily stool sample results - $r(t) = \frac{T^{+}(t)}{T(t)}$.

(b) The proportions and 95% CI of the weekly smoothed stool samples data - $\tilde{r}(t) = \frac{\sum_{t'=t-3}^{t+3} T^{+}(t')}{\sum_{t'=t-3}^{t+3} T(t')}$.

The confidence intervals related to the daily data are huge and easily cover the estimated WPV1 prevalence range. Even the smaller intervals given by the weekly smoothed data cover the range of WPV1 prevalence given by the model almost completely.

6. Results of model with no seasonal variation in transmission

In order to compare the results of the model with seasonal variation in transmission to the results of a model without seasonality, we ran the MCMC procedure for a second time while fixing the seasonality parameters so that $\delta = 0$ (i.e., no seasonal variation) and $\phi = \bar{\phi}$ (i.e., the mean of the peak time in ω (eq. S4)). While the latter has no effect on the results of the model fitting (with $\delta = 0$ the value of ϕ does not affect the transmission rates), we did so in order to obtain the maximum value of the prior distribution for ϕ, so as to allow a comparison of the likelihoods obtained with this model and the model with seasonality (see table S2 below). Table S1 and figure S8 show the results obtained using this model. The mean and 95% CI estimates obtained using this model for the period of infectiousness (d_I) and for the reproductive number ($\bar{R}$) are similar to the estimates obtained using the model with seasonality (compare to table 2 in the main text). The posterior distribution for the per-dose efficacy of OPV (ρ) in this model looks similar to its prior distribution (fig. S8c), while the posterior for the initiation time of the epidemic (t_0) is tilted towards the earlier times in the considered range (fig. S8d). The main difference between the two models is that without seasonality, the model does not project the possibility of another epidemic wave during 2014 and beyond, in case the OPV campaign had not taken place (compare figures S8e,g to figures 4a,c in the main text). Otherwise most of the parameter values are in the same range (table 2 and table S1).

**output**	**mean [95% CI]**
reproductive number	1.79 [1.49-2.45]
mean duration of the infectious period	15.6 days [9.0-24.3]
per-dose efficacy of OPV	0.56 [0-1]
attack rate with the OPV campaign at the end of 2014	0.47 [0.26-0.67]
attack rate without the OPV campaign at the end of 2014	0.63 [0.49-0.76]
reduction in attack rate due to OPV campaign	0.16 [0-0.39]
end time of the outbreak with the OPV campaign	April 23, 2014 [Jan. 28, 2014 - Sep. 16, 2014]
end time of the outbreak without the OPV campaign	Sep. 17, 2014 [Mar. 22, 2014 –Apr. 18, 2015]
reduction in outbreak duration due to OPV campaign	146 days [0-305]

Table S1: Parameter estimates of model fitting using a model with no seasonality.

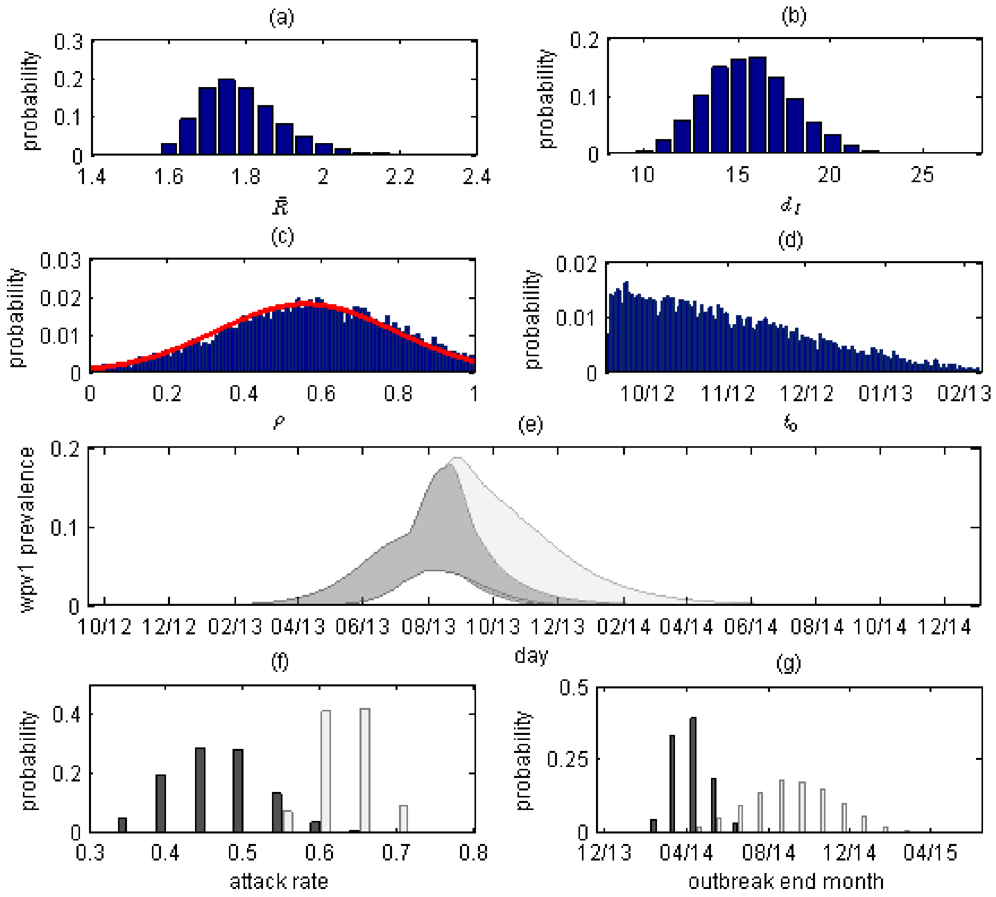

Figure S8: Outcomes of fitting a model without seasonal variation in the transmission.

(a) Posterior distribution for the reproductive number.

(b) Posterior distribution for the mean infectious period.

(c) Posterior distribution for the per-dose efficacy of OPV. Red curve is showing the prior distribution used for this parameter.

(d) Posterior distribution for the start time of the outbreak.

(e) 95% credible intervals of WPV1 prevalence with the OPV campaign (dark grey) and without the OPV campaign (light grey).

(f) The posterior distribution of the overall attack rate at the end of 2014 with (dark grey bars) and without (light grey bars) the OPV campaign.

(g) The posterior distribution for the end time of the outbreak showing the probability of the outbreak ending on a particular month with (dark grey bars) and without (light grey bars) the OPV campaign.

	model with seasonality	model without seasonality
$D(\bar{\theta}) = -2 \cdot log(L(\bar{\theta}))$	185.20	187.13
$\overline{D} = -2 \cdot E\left(log\big(L(\theta)\big)\right)$	188.51	189.77
$p_{D1} = \overline{D} - D(\bar{\theta})$	3.31	2.64
$p_{D2} = \frac{1}{2}\widehat{Var}(D(\theta))$	3.88	3.06
$DIC_1 = p_{D1} + \overline{D}$	191.82	192.41
$DIC_2 = p_{D2} + \overline{D}$	192.39	192.83

Table S2: A comparison of the two models, with and without seasonality, using deviance information criterion (DIC) [17]. $\bar{\theta}$ was calculated as the average of the sampled values obtained using the MCMC. $\overline{D}$ was calculated using the mean of the log-likelihoods of all the sampled values and p_{D2} was calculated using the variance of the log-likelihoods of all the sampled values. The model with seasonality, which incorporates two additional parameters compared to the non-seasonal model, manages to obtain slightly better likelihoods over all, as can be seen by the difference in the value of $\overline{D}$ between the two models. However, the calculated effective number of parameters (either p_{D1} or p_{D2}) is higher for this model so that the obtained DIC score (either DIC_1 or DIC_2) are only slightly better for the seasonal model, and not enough to rule out the non-seasonal model.

7. Results of inference without the limitation set using ES

Table S3 and figures S9 and S10, summarize the results obtained by the model fitting when removing the limitation set using the ES data (eq. S7). Without the limitation the sampled posteriors include samples with longer mean durations of infection and larger amplitudes, leading to more uncertainty in the estimated outcomes of the outbreak and the possibility of a second small epidemic wave during 2014 and 2015 even after the OPV vaccinations. In this case, without the OPV vaccinations the projected epidemic wave during 2014 could have been huge (fig. S11a).

**output**	**mean [95% CI]**
mean reproductive number	1.81 [1.48-2.43]
mean duration of the infectious period	19.9 days [9.2-40.3]
amplitude of seasonal variation in transmission	0.95 [0-2.38]
peak day of seasonal variation in transmission	128 [71-198]<br>May 8 [Mar. 12 – Jul. 17]
per-dose efficacy of OPV	0.61 [0-1]
attack rate with the OPV campaign<br>at the end of 2014	0.35 [0.14-0.65]
attack rate without the OPV campaign<br>at the end of 2014	0.59 [0.29-0.90]
reduction in attack rate due to OPV campaign	0.24 [0-0.73]
end time of the outbreak with the OPV campaign	July 19, 2014<br>[Jan. 27, 2014 - Feb. 1, 2016]
end time of the outbreak without the OPV campaign	July 20, 2015<br>[Feb. 18, 2014 - Dec. 31, 2016]
reduction in outbreak duration due to OPV campaign	365 days [-134-895]

Table S3: Results of the model fitting obtained without imposing the condition based on the ES data (compare to the results with the inclusion of the limitation in table 2 of the main text).

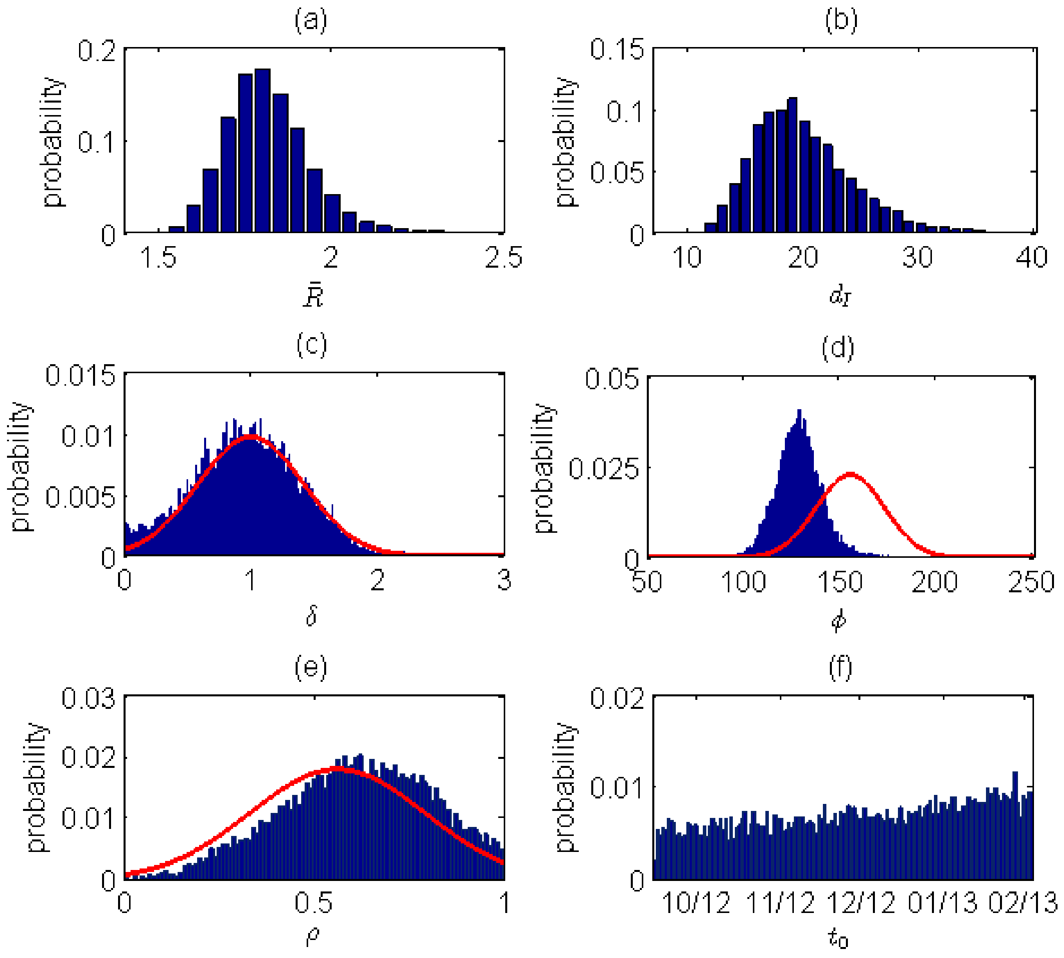

Figure S9: Posterior distributions for the model parameters obtained from the MCMC sampling without imposing the condition based on the ES data (compare to the results with the inclusion of the limitation in figure 2 of the main text):

(a) Posterior distribution for the mean reproductive number.

(b) Posterior distribution for the mean infectious period.

(c) Posterior distribution for the amplitude of seasonal variation in transmission. The red curve shows the used prior distribution.

(d) Posterior distribution for the peak time of seasonal variation in transmission. The red curve shows the used prior distribution.

(e) Posterior distribution for the per-dose efficacy of OPV. The red curve shows the used prior distribution.

(f) Posterior distribution for the start time of the outbreak.

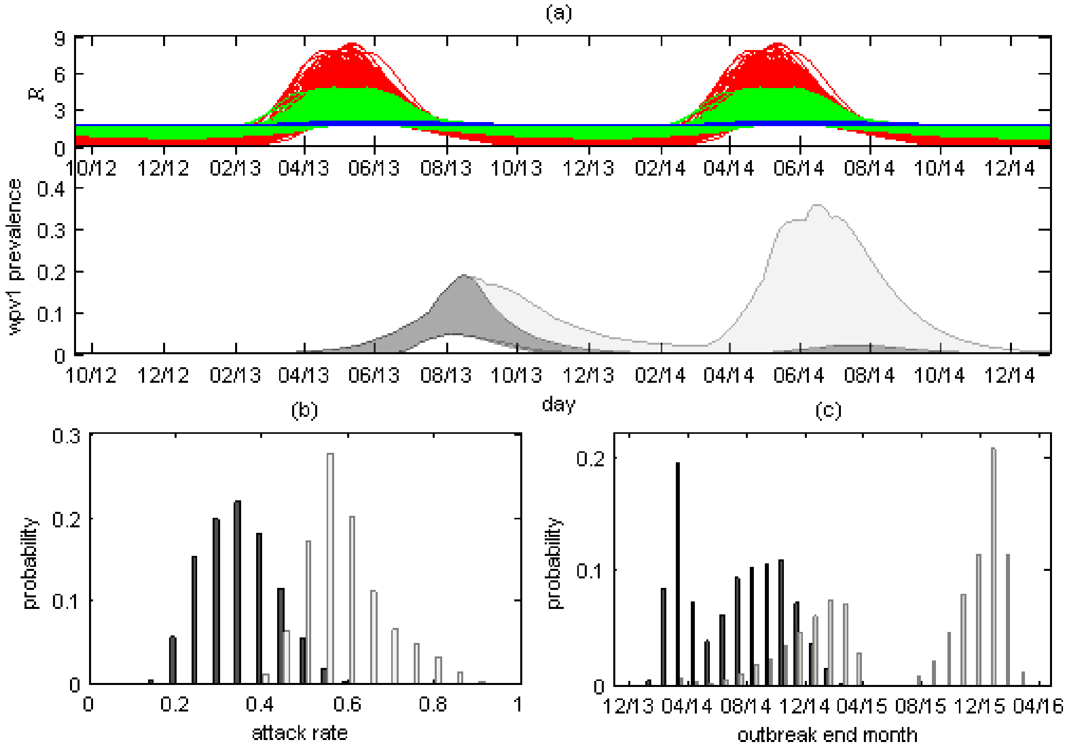

Figure S10: Outcomes of model fitting without imposing the condition based on the ES data (compare to the results with the inclusion of the limitation in figure 4 of the main text):

(a) **Top panel**: 1000 plots of the value of the reproductive number (R) in time, calculated using eq. S2 and S4 with 1000 values of $\bar{R}$, δ and ϕ, randomly sampled out of the values obtained by the MCMC. Blue curves showing results for $\delta \leq 0.1$, red curves showing results for $\delta \geq 1$ and green curves showing results for everything in between.

Bottom panel: 95% CI of WPV1 prevalence with the OPV campaign (dark grey) and without the OPV campaign (light grey). Without the limitation using ES we obtain the possibility of a small second wave during 2014 with the OPV campaign and a huge second wave without the OPV campaign.

(b) The posterior distribution of the overall attack rates at the end of 2014 with (dark grey bars) and without (light grey bars) the OPV campaign.

(c) The posterior distribution for the end time of the outbreak showing the probability of the outbreak ending on a particular month with (dark grey bars) and without (light grey bars) the OPV campaign.

8. Posterior distributions of the outbreak end time when using a threshold of ten infected individuals for the epidemic extinction

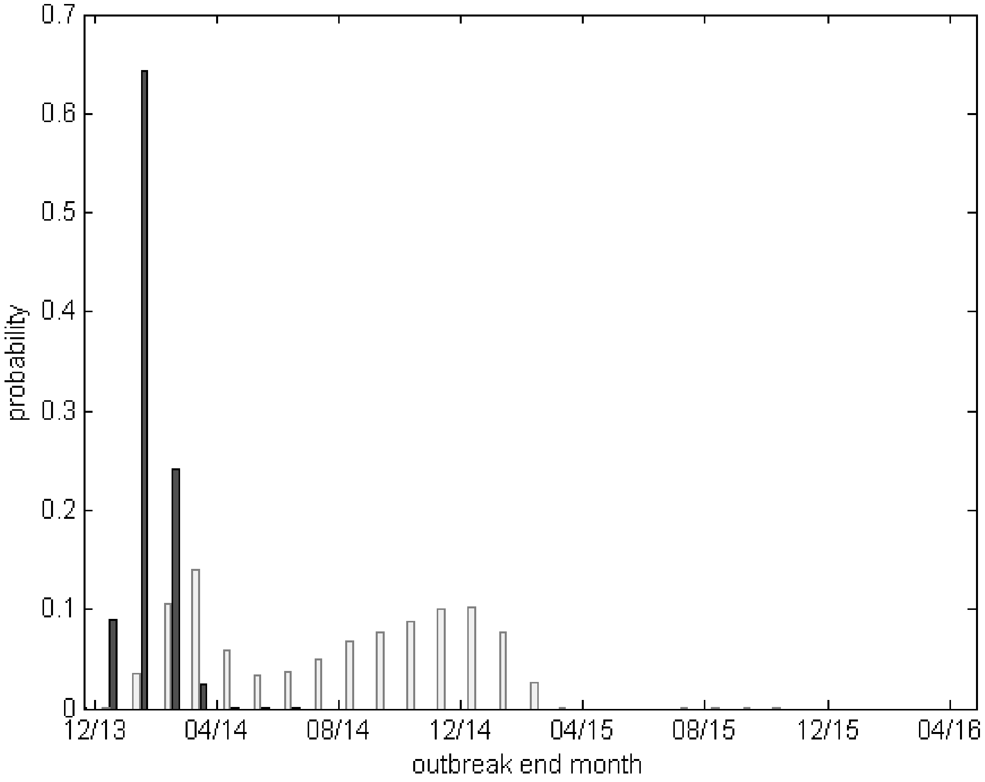

Figure S11: The posterior distribution of the end time of the outbreak using the full model (including seasonality) with the OPV campaign (dark grey bars) and without it (light grey bars), while setting the threshold for the epidemic extinction to ten infected individuals. In this case, according to the figure, there is considerable probability that without the OPV campaign the outbreak would have lasted up until February 2015, and only a very small probability that it would have lasted for more than that. This is in contrast to the results obtained when using a threshold of one infected individual which shows substantial probability for the outbreak without the OPV vaccinations lasting until March 2016 (see fig. 4c in the main text). This indicates that in a model incorporating stochasticity in the transmission of WPV, an epidemic wave after 2014 may not occur, due to stochastic fade-out of the outbreak during the period of low transmissibility, whereas the epidemic wave during 2014 would most likely occur in a stochastic model as well.

9. Epidemic curves with different vaccination starting dates

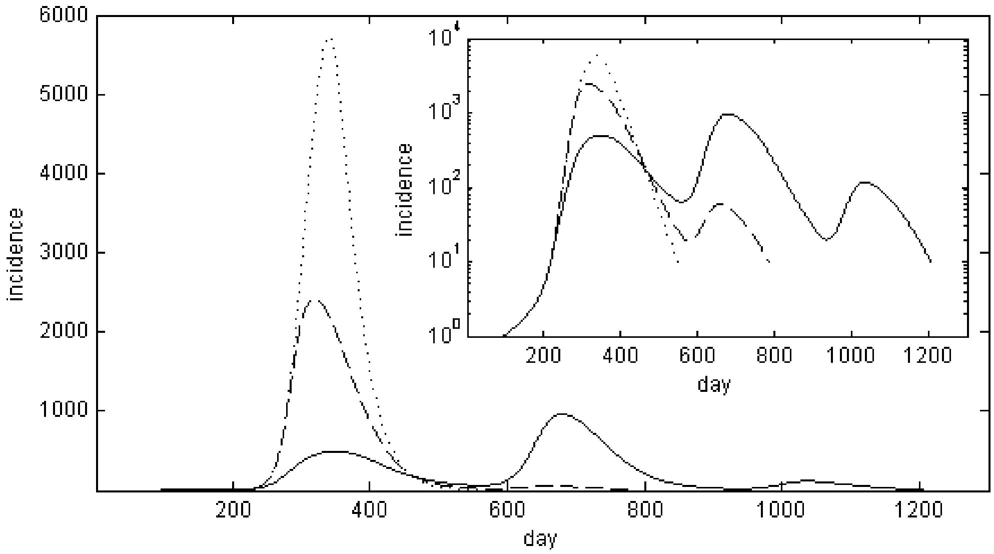

Figure S12: The epidemic curves obtained from running the transmission model using the mean estimates obtained from the MCMC sampling (table 2 of the main text) with an OPV campaign of one dose covering 50% of the population under three scenarios: vaccination beginning on April 5, 2013 (solid line), June 5, 2013 (dashed line) or August 5, 2013 (dotted line) - the three considered starting dates used in figure 5 of the main text. As in the preparation of figure 5, the simulations of the model were run until the incidence falls below ten infected individuals, in order to take into account the probability of a stochastic fade-out of the outbreak during the periods of low transmissibility. The Y-axis is showing the incidence of infected cases. The inset shows the same plot with the y-axis plotted in log-scale, allowing a closer examination of the tail end of the epidemic. As can be seen, starting the vaccination earlier reduces the magnitude of the epidemic at the cost of prolonging the duration of the outbreak.

10. References

1. Wright PF, Wieland-Alter W, Ilyushina N a, Hoen AG, Arita M, et al. (2014) Intestinal immunity is a determinant of clearance of poliovirus after oral vaccination. J Infect Dis 209: 1628–1634. doi:10.1093/infdis/jit671.

2. Duintjer Tebbens RJ, Pallansch M a, Kew OM, Cáceres VM, Sutter RW, et al. (2005) A dynamic model of poliomyelitis outbreaks: learning from the past to help inform the future. Am J Epidemiol 162: 358–372. doi:10.1093/aje/kwi206.

3. Blake IM, Martin R, Goel A, Khetsuriani N, Everts J, et al. (2014) The role of older children and adults in wild poliovirus transmission. Proc Natl Acad Sci U S A 1: 10604–10609. doi:10.1073/pnas.1323688111.

4. Kalkowska DA, Tebbens RJD, Grotto I, Shulman LM, Anis E, et al. (2015) Modeling Options to Manage Type 1 Wild Poliovirus Imported Into Israel in 2013. J Infect Dis 211: 1800–1812. doi:10.1093/infdis/jiu674.

5. Martinez-bakker M, King AA, Rohani P (2015) Unraveling the Transmission Ecology of Polio. PLoS Biol 13: 1–21. doi:10.1371/journal.pbio.1002172.

6. Hatch LA, Hughes KE, Pilfold JN (1958) Observations on the excretion of type 1 poliovirus from cases and their contacts. Am J Med Sci 236: 419–424.

7. Gelfand HM, Fox JP, Leblanc DR, Elveback L (1960) Studies on the development of natural immunity to poliomyelitis in Louisiana. V. Passive transfer of polioantibody from mother to fetus, and natural decline and disappearance of antibody in the infant. J Immunol 85: 46–55.

8. Alexander JP, Gary HE, Pallansch MA (1997) Duration of poliovirus excretion and its implications for acute flaccid paralysis surveillance: a review of the literature. J Infect Dis 175Suppl1: S176–S182.

9. Ghendon YZ, Sanakoyeva II (1961) Comparison of the Resistance of the Intestinal Tract to Poliomyelitis virus (Sabin's Strains) in Persons after Naturally and Experimentally Acquired Immunity. Acta Virol 5: 265–273.

10. Laassri M, Lottenbach K, Belshe R, Wolff M, Rennels M, et al. (2005) Effect of Different Vaccination Schedules on Excretion of Oral Poliovirus Vaccine Strains. 20852.

11. Mangal TD, Aylward RB, Grassly NC (2013) The potential impact of routine immunization with inactivated poliovirus vaccine on wild-type or vaccine-derived poliovirus outbreaks in a posteradication setting. Am J Epidemiol 178: 1579–1587. doi:10.1093/aje/kwt203.

12. Patriarca P a, Sutter RW, Oostvogel PM (1997) Outbreaks of paralytic poliomyelitis, 1976-1995. J Infect Dis 175 Suppl : S165–S172.

13. Behrend MR, Hu H, Nigmatulina KR, Eckhoff P (2014) A quantitative survey of the literature on

poliovirus infection and immunity. Int J Infect Dis 18: 4–13. doi:10.1016/j.ijid.2013.09.005.

14. Shulman L, Gavrilin E, Jorba J, Martin J, Burns C, et al. (2014) Molecular epidemiology of silent introduction and sustained transmission of wild poliovirus type 1, Israel, 2013. Eurosurveillance 19: 20709. doi:10.2807/1560-7917.ES2014.19.7.20709.

15. Y. Manor et al. (2014) Intensified environmental surveillance supporting the response to wild poliovirus type 1 silent circulation in Israel. Euro surveil 19: 20708.

16. Neal RM (2003) Slice Sampling. Ann Stat 31: 705–767.

17. Spiegelhalter DJ, Best NG, Carlin BP, van der Linde A (2002) Bayesian measures of model complexity and fit. J R Stat Soc Ser B (Statistical Methodol (Statistics Soc 64: 583–639.
